# Inferring cell diversity in single cell data using consortium-scale epigenetic data as a biological anchor for cell identity

**DOI:** 10.1093/nar/gkad307

**Published:** 2023-05-01

**Authors:** Yuliangzi Sun, Woo Jun Shim, Sophie Shen, Enakshi Sinniah, Duy Pham, Zezhuo Su, Dalia Mizikovsky, Melanie D White, Joshua W K Ho, Quan Nguyen, Mikael Bodén, Nathan J Palpant

**Affiliations:** Institute for Molecular Bioscience, The University of Queensland, Brisbane, QLD, Australia; Institute for Molecular Bioscience, The University of Queensland, Brisbane, QLD, Australia; Institute for Molecular Bioscience, The University of Queensland, Brisbane, QLD, Australia; Institute for Molecular Bioscience, The University of Queensland, Brisbane, QLD, Australia; Institute for Molecular Bioscience, The University of Queensland, Brisbane, QLD, Australia; School of Biomedical Sciences, Li Ka Shing Faculty of Medicine, The University of Hong Kong, Pokfulam, Hong Kong SAR, China; Laboratory of Data Discovery for Health Limited (D24H), Hong Kong Science Park, Hong Kong SAR, China; Institute for Molecular Bioscience, The University of Queensland, Brisbane, QLD, Australia; Institute for Molecular Bioscience, The University of Queensland, Brisbane, QLD, Australia; School of Biomedical Sciences, Li Ka Shing Faculty of Medicine, The University of Hong Kong, Pokfulam, Hong Kong SAR, China; Laboratory of Data Discovery for Health Limited (D24H), Hong Kong Science Park, Hong Kong SAR, China; Institute for Molecular Bioscience, The University of Queensland, Brisbane, QLD, Australia; School of Chemistry and Molecular Biosciences, The University of Queensland, Brisbane, QLD, Australia; Institute for Molecular Bioscience, The University of Queensland, Brisbane, QLD, Australia

## Abstract

Methods for cell clustering and gene expression from single-cell RNA sequencing (scRNA-seq) data are essential for biological interpretation of cell processes. Here, we present TRIAGE-Cluster which uses genome-wide epigenetic data from diverse bio-samples to identify genes demarcating cell diversity in scRNA-seq data. By integrating patterns of repressive chromatin deposited across diverse cell types with weighted density estimation, TRIAGE-Cluster determines cell type clusters in a 2D UMAP space. We then present TRIAGE-ParseR, a machine learning method which evaluates gene expression rank lists to define gene groups governing the identity and function of cell types. We demonstrate the utility of this two-step approach using atlases of *in vivo* and *in vitro* cell diversification and organogenesis. We also provide a web accessible dashboard for analysis and download of data and software. Collectively, genome-wide epigenetic repression provides a versatile strategy to define cell diversity and study gene regulation of scRNA-seq data.

## INTRODUCTION

Single-cell RNA sequencing offers unprecedented opportunities to investigate the diversity of cell processes underpinning development and disease. Standard analysis workflows typically involve stepwise analytic tools that decompose high-dimensional data to reveal the complexity of and relationships between cell types. However, the computational challenges associated with single cell analysis are considerable, partially due to common user-defined parameter settings that make interpretation of cell diversity a subjective exercise. Furthermore, downstream analyses often rely on prior knowledge, using known marker genes, subjective data subtraction and reference annotation databases (1). Currently, few methods utilize unsupervised orthogonal reference points for defining cellular diversity and interpreting genetic regulation of cell states.

Cell type clustering methods are essential for downstream analyses to generate hypotheses about cellular processes. Most current clustering algorithms group cells based on gene expression similarity and rely on mathematically defined constraints for data analysis (2). Agglomerative clustering methods such as SINCERA (3), pcaReduce (4), and CIDR (5) use distance measurements such as Euclidean, Manhattan, Jaccard, Minkowski and Canberra (6) to merge clusters iteratively based on similarities (7). K-means clustering groups data points by taking a distance measure and iteratively optimising the centroid of each group to minimize within-cluster distance (8). Model-based approaches like Gaussian Mixture Models (GMM) exemplified by scGMAI (9) use (multiple) distributions to capture relationships between cells (8). Density-based clustering such as DBSCAN (10) and GiniClust (11), are non-parametric approaches that group cells based on data point density, where low-density areas are considered as outliers. Lastly, graph-based clustering is an extension of density-based clustering (12) where relationships among cells are represented by a similarity graph (13). Popular clustering approaches, such as Louvain and Leiden, are implemented in Seurat (14) and SCANPY (15).

Owing to the complex nature of cell clustering, there is no widely accepted standard for parameter setting or method selection (2). Moreover, most clustering methods assume all cells in a dataset are equally important and therefore interpret them equally, despite the well-known influence of sequencing depth, ambient RNA and technical variation on the variability and accuracy of scRNA-seq data (16–18). Establishing justifiable and generalisable criteria for excluding data beyond the standard trimming of doublets and high-quality cells remains challenging.

Similarly, gene expression analysis often depends on arbitrary selection of computational parameters and reference points. For instance, in differential expression (DE) gene analysis, using highly variable genes (19) between cell clusters can results in data interpretation that is only relevant to individual datasets (20). Grouping genes with co-expression patterns has been an effective strategy to study functional gene-to-gene relationship. For instance, gene modules identified by fitting a Gaussian mixture model to gene expression data has been used to stratify patient (21–23) and various cancer cell subtypes (24–27) by inferring functional relationship between genomic elements. Also, Weighted correlation network analysis (WGCNA) connects co-expressed gene modules to external resources such as clinical data, SNPs (single-nucleotide polymorphisms) and Gene Ontology (GO) (28). However, current models mostly rely on expression data alone, and the biologically meaningful incorporation of orthogonal genomic data into computational systems biology is not commonly used. Alternative methods also use cell-level abundance features to identify cell clusters (29), or model distributional differences in the expression of individual genes to extract biological meaning from the regulatory network relevant to cell clusters (30–34). These methods may lack more complex gene regulatory information needed to interpret gene expression networks and are often heavily influenced by the data structure (33,35).

Recent studies, such as CADD (36) and UnTANGLeD (37), use statistical methods to quantify probabilities and relationships in the genome to help interpret complex genomic data. These methods are powerful because they provide an independent, unsupervised, simple and quantifiable analysis of the genome that seamlessly interfaces with orthogonal genomic data to interpret genetic information. We have recently developed TRIAGE (Transcriptional Regulatory Inference Analysis of Gene Expression), a computational method that provides an independent and unsupervised model to interpret genomic data from any cell or tissue type, without the need for external reference data, statistical cutoffs, or prior knowledge. We used consortium data from the Human Cell Atlas, FANTOM and a draft map of the human proteome to demonstrate that histone modification deposition patterns can be used to discern genes underpinning cell identity (20). We found that genes frequently harboring broad H3K27me3-domains across diverse cell types significantly enrich for cell-type specific regulatory genes (20). A genome-wide quantitative feature devised from TRIAGE, called ‘repressive tendency score’ (RTS), serves as an independent reference point that can infer cell-type regulatory potential for each protein-coding gene in different cell types, which could help advance our understanding of gene regulation and its impact on cell identity.

Here, we present a two-step pipeline that complements the baseline methods for identifying transcriptionally distinct cell populations. The pipeline draws on the fundamental principles of TRIAGE, enabling the analysis of cell clustering and interpretation of cell populations. This approach is scalable to any cell type and data type, including applications across species, making it possible to analyse biological diversity in scRNA-seq data.

## MATERIALS AND METHODS

### Identifying priority cell type-specific regulatory genes

To calculate the RTS, we followed the two-step approach described in TRIAGE (20), using EpiMap data from 834 cell types [39]: (i) we calculated the total length (breadth) of H3K27me3 domains in base pairs for each gene across 834 EpiMap cell types, and (ii) we multiplied each value by the proportion of cell types in which the gene's H3K27me3 breadth was among the top 5% of broad domains. This analysis assigned a single score to each gene, indicating its association with broad H3K27me3 domains. The genes were ranked by their RTS, and a priority set of 993 genes were identified as those above the inflection point.

### Correlation analysis of RTS priority gene expression during cell differentiation pseudotime

To identify genes that significantly influence differentiation potential, we used a single-cell RNA sequencing dataset characterising definitive endoderm differentiation using 125 iPSC lines (38). For each cell line, we calculated the average expression of each RTS priority gene at day 0, day 1 and day 2 of endoderm differentiation. Next, we performed a linear regression between the average gene expression at pluripotency and the average pseudotime of each cell line at day 3 of differentiation, as calculated in the original data (38). We applied a false discovery rate correction to account for multiple testing. We repeated this analysis for gene expression at days 1 and 2 of differentiation.

### Evaluation of relationship between RTS priority gene rank and differentiation time.

To investigate the correlation between RTS values and lineage development, as well as cell type identification, we analysed a time-course *in vitro* cardiac cell differentiation (39). We examined the relationship between RTS values and cell development lineages by tracking the distribution of RTS values across different stages of differentiation. We also calculated the average expression level of each RTS priority gene at each stage of differentiation compared to its corresponding RTS value and visualised results using *pheatmap* R package (40) with *Z*-score normalisation for each gene.

### Pre-processing of mouse gastrulation atlas data (41)

To prepare the mouse gastrulation atlas data, we converted mouse gene names to human gene names and removed genes that had no expression in all cells. Additionally, we excluded low-quality cells using the *perCellQCMetrics* and *quickPerCellQC* functions from the R package *scater* (42). The filtered data was then normalised by library size using the *computeSumFactors* function from the *scran* R package (43). Normalised counts were used for *TRIAGE* (20) conversion, which generates a discordance score (DS) count matrix. To reduce the dimensionality of the data, we performed a downstream principal component analysis (PCA) using the *RunPCA* function in *Seurat* and computed 50 principal components (PCs). We then used the 50 PCs for UMAP (uniform manifold approximation and projection) and t-SNE (t-distributed stochastic neighbour embedding) through the *RunUMAP* and *Runtsne* function (14). All other data used in this paper were processed in the same way as the mouse gastrulation atlas data. Given that UMAP has demonstrated its usefulness in identifying cell types in highly heterogeneous datasets, as well as its scalability and faster runtime in scRNA-seq data (44–49), we selected UMAP as the dimensional reduction space to apply our TRIAGE-Cluster pipeline. However, we also compared the performance of UMAP and t-SNE with respect to our pipeline. The *FindClusters* function with Louvain algorithm as default setting is utilized in Seurat to obtain clusters at 20 different resolutions ranging from 0 to 2 with an increment of 0.1. These clusters are then utilized in downstream benchmarking analysis.

### Development of TRIAGE-cluster

#### Density estimation and peak selection

For each cell in the data set, the RTS priority gene with highest expression for each cell was identified and its corresponding RTS was used for downstream analysis as follows: UMAP coordinates were used with the assigned RTS for each cell as weight for estimating kernel density. The function *stats.gaussian_kde* from *scipy* package (50) estimates the probability density function of RTS-weighted cell states. For any given cell $x$, the weighted Gaussian kernel density estimator $\hat{f}$can be calculated as


$$\begin{equation*}\hat{f}\left( x \right) = \frac{1}{{nh}}\mathop \sum \limits_{i\ = \ 1}^n {w}_iK\left( {\frac{{x - {x}_i}}{h}} \right)\end{equation*}$$

where $n$ is the total number of cells, ${w}_i$ is RTS and ${x}_i$ is the UMAP embeddings of the $i$-th cell, $h$ is the bandwidth of a Gaussian kernel $K$ defined as


$$\begin{equation*}K\left( \mu \right) = \frac{1}{{\sqrt {2{\rm{\pi }}{{\rm{\sigma }}}^2} }}\exp \left( { - \frac{{{\mu }^2}}{{2{{\rm{\sigma }}}^2}}} \right)\end{equation*}$$

where $\mu$ is the mean of the Gaussian distribution and $\sigma$ is its standard deviation.

Bandwidth $h$ was determined using Scott's Rule (51) and adjusted to suit scRNAseq data as follows


$$\begin{equation*}h\ = \ 0.3*\ {n}^{\left( { - \frac{1}{{d + 4}}} \right)}\end{equation*}$$

where $n$ denotes number of cell and $d$ denotes number of dimensions. A density map with contours (contour levels = 10) were generated. At each contour level, we used unsupervised *DBSCAN* from the *sklearn* package (52) to group cells into spatially separated clusters. From each contour level, we selected regions with local RTS maxima as peaks (i.e. clusters without smaller clusters within them from a higher contour level). Peaks (i.e. clusters) from all contour levels represent the cell population diversity in the dataset. It should be noted that UMAP coordinates are not linear transformations of input data and only preserve close similarities (53). We hypothesised this is sufficient in order for density estimates to work and tested it against another topological dimension reduction method, t-SNE, by comparing the biological diversity capture from peaks obtained from UMAP and t-SNE.

#### Jaccard similarity dendrogram

We computed the Euclidean distance matrix using the Jaccard similarities of the top 100 DS genes for each pair of peaks, and then used the R package *hclust* to perform hierarchical clustering and extract the dendrogram representation of relationship between all peaks.

#### Adjusted rand index (ARI) analysis

We used the ARI (54) to assess the performance of different clustering methods by comparing it with the original annotation of the mouse gastrulation atlas data (41). Additionally, to evaluate the impact of removing RTS priority gene on accuracy of data structure, we employed *adjustedRandIndex* function from *mclust* R package (55), to compare four removal scenarios with the gold standard that we used, which was the high expression RTS genes for all cells generated from the original RTS priority gene rank. We tested four gene removal scenarios: (i) random removal of genes from the RTS priority gene list, (ii) removal of RTS priority genes from the bottom rank to the top, (iii) removal of RTS priority genes from the top rank to the bottom and (iv) random removal of genes from the whole genome. Each removal step is 50 genes.

#### Spearman rank correlation

To assess the ability of TRIAGE-Cluster and Seurat in identifying biologically distinct cell types, we (i) evaluated peak-peak correlation across multiple resolutions using the average expression of either the entire transcriptome (16 946 genes) or a set of highly variable genes (1000 genes) with both the original expression matrix and the TRIAGE-converted matrix (DS matrix), and (ii) assessed peak-peak correlation with selected resolutions giving the same number of clusters for both methods, using the entire transcriptome (16 946 genes) with the DS matrix as it provides comprehensive understanding of the biological context. We conducted Spearman rank correlation analysis using the *cor* function from the *stats* package. Highly variable genes are defined using the *modelGeneVar* and *getTopHVGs* (*n* = 1000) functions from the *scran* package.

#### Gene ontology enrichment

To verify that TRIAGE-Cluster peaks capture a greater biology diversity, we compared the significant GO terms (*P*-value < 0.05) between TRIAGE-Cluster peaks and Seurat clusters, using the top 100 genes ranked by DS from each peak or cluster. We conducted a GO enrichment analysis using the *topGOtable* function from the *pcaExplorer* package with *org.Hs.eg.db*, the genome wide annotation for human.

#### SC3 (single-cell consensus clustering) analysis

Since the mouse gastrulation atlas data includes over 5000 cells, we employed a hybrid approach with an SVM model to obtain clusters as outlined in the SC3 pipeline (56). We generated the same number of clusters as TRIAGE-Cluster for downstream benchmarking analysis. Additionally, to assess cell diversity captured by SC3 clusters, we performed Spearman rank correlation and GO enrichment using the same cluster numbers used for benchmarking with Seurat as examples.

#### MAGIC imputation

To evaluate the impact of sparsity of scRNA-seq data on RTS priority genes and peaks, we employed the *magic* function with default settings from the *MAGIC* R package (57) to restore the structure and minimize the ‘dropout’ issue in the mouse gastrulation atlas data. Subsequently, we compared the peaks generated from the MAGIC-imputed data with those from the original mouse gastrulation atlas data to assess biological diversity by GO enrichment analysis.

### Development of TRIAGE-ParseR

#### Processing of H3K27me3 data

We used consolidated H3K27me3 BED files for 111 Roadmap tissue and cell types (58). We also downloaded bigwig files representing 834 samples from EpiMap (59) and converted them into *bedgraph* format using *bigWigToBedGraph* (60,61). Subsequently, we used *MACS2* (62) to call H3K27me3 enriched loci with -broad option to capture broad deposition of H3K27me3.

#### Extracting H3K27me3 patterns

We performed PCA to extract orthogonal patterns of H3K27me3 depositions from consortium-level epigenomic data (58,59). H3K27me3 deposition values were defined by breadth of H3K27me3 peaks overlapping proximal regions of RefSeq annotated transcription start sites (TSS) (±2.5 kb) of genes. If multiple peaks overlap a given gene, the broadest peak was chosen. We used top 67 (Roadmap) or 60 (EpiMap) top PCs which explain most of the data variance (i.e. 96.5% or 97.7% respectively). PCA loading values represent unit scale components for covariances between observed H3K27me3 breadths of different samples while eigen vectors indicate degree of gene's correlation to H3K27me3 patterns.

#### Analysis of H3K27me3 patterns among top 100 genes ranked by DS

We compared underlying H3K27me3 patterns associated with top 100 genes ranked by DS which are highly enriched with variably expressed transcription factors between different tissue groups (defined by expression coefficient of variation > 1). To this end, we first analysed enrichment of H3K27me3 patterns across genes. For each gene, empirical probability of observing a given value higher than the 95th percentile of the 26 827 RefSeq genes in each eigen vector was calculated. We define that genes with the empirical *P*-value of <0.05 (right-tail) demonstrate strong association with a given H3K27me3 pattern. To understand whether each of the top 100 genes ranked by DS demonstrate significant association with a given pattern, we randomly sampled another 100 genes (null distribution) and counted incidences (*r*) where the observed value is higher than samples in the null distribution. We permuted this process 1000 times and calculated a probability of gene *i* (${p}_i$) to have a value higher than 95% of the random samples.


$$\begin{equation*}{p}_i = \frac{{{r}_i + 1}}{{1000 + 1}}\end{equation*}$$

#### Identifying an appropriate cluster number for genes by H3K27me3 patterns

To cluster genes ranked by DS based on associated H3K27me3 patterns, we used Bayesian information criterion (63) to find a suitable number of clusters. The BIC finds a suitable number of clusters from distributions of observed H3K27me3 depositions. While we used top 100 genes ranked by DS based on their enrichment of regulatory genes, the user can select any meaningful number of genes for this analysis. For a given top 100 TRIAGE-prioritised gene set (i.e. *n* = 100), values of top *m* PCs (i.e. ${X}_i = {x}_{i,1},{x}_{i,2}, \ldots ,{x}_{i,m}$, for *i*-th ranked gene where $i \in \{ {1,2, \ldots ,100} \}$) were used as features for the parameter selection. The method then ranks PCs by level of variance observed among the input gene set and selects top *m* PCs (default = 10 PCs). Subsequently, the maximum likelihood to observe PC values from all top 100 genes ranked by DS was iteratively calculated with a varying number of clusters $\theta$ where $\theta \in \{ {1,2, \ldots ,100} \}$. The cluster was defined by a Gaussian model ${\varphi }_j(X|{\mu }_{j,1},{\mu }_{j,2}, \ldots ,{\mu }_{j,m},{\rm{\ }}{\sigma }_{j,1},{\sigma }_{j,2},{\rm{\ }} \ldots ,{\rm{\ }}{\sigma }_{j,m})$ for *j*th cluster centred at means ${\mu }_j$with variances ${\sigma }_j$ across *m* PCs, where *m* is 67 or 70 for Roadmap or EpiMap data respectively. The clusters represent a region of high probability mass estimated by expectation-maximisation (EM) algorithm. Intuitively, if $\theta$ is 1, every gene belongs to the same cluster while if $\theta$ is 100, every gene belongs to its own cluster. The aim of BIC is to find an appropriate value for $\theta$ that gives the lowest BIC value (i.e. a cluster number which is the most balanced point between model complexity and likelihood of observing data given the cluster number and model parameters).


$$\begin{equation*}BIC\ = {\rm{\ }}\theta \cdot \ln n - 2 \cdot \ln \hat{L}\end{equation*}$$

where $\hat{L}$ is the maximum value of the likelihood function (L) given the number of clusters ($\theta$) and model parameters. The likelihood functions are written as follows:


$$\begin{equation*}L\left( {\theta {\rm{|}}{X}_1, \ldots ,{\rm{\ }}{X}_{100}} \right) = \mathop \prod \limits_{i = 1}^{100} \cdot \mathop \sum \limits_{k = 1}^\theta {\pi }_k \cdot N\left( {{x}_i;{\mu }_k,{\sigma }^2_k} \right)\end{equation*}$$

where ${\pi }_k$represents a probability (or mixture proportion) that gene ${X}_i$ belonging to *k*-th cluster:


$$\begin{equation*}\ln \left( {L\left( {\theta {\rm{|}}{X}_1, \ldots ,{\rm{\ }}{X}_{100}} \right)} \right) = \mathop \sum \limits_{i = 1}^{100} \ln (\mathop \sum \limits_{k = 1}^\theta {\pi }_k \cdot N\left( {{x}_i;{\mu }_k,{\sigma }^2_k} \right))\end{equation*}$$

We aim to find $\theta$ such that the BIC is the smallest. Hence, this problem is equivalent to finding the maximum log-likelihood value given $\theta$ (i.e. $\ln \hat{L}$).


$$\begin{equation*}h = argma{x}_\theta \ln (L(\theta |{X}_1, \ldots ,{\rm{\ }}{X}_{100}))\end{equation*}$$

Due to sensitivity of EM method to initial parameter values, this analysis was repeated multiple times (default = 10 times) and the most frequently occurring values were chosen as an appropriate number of clusters. To ensure reproducibility of the results, random state parameter for GaussianMixture function from scikit-learn Python module was set to 42.

#### Assigning genes to H3K27me3 clusters using gaussian mixture models

After identifying an appropriate number of clusters ($h$) for a given prioritised gene set, the next step is to probabilistically assign each gene into one of the clusters. We assumed multivariate Gaussian distributions for each cluster. The Gaussian Mixture Model (GMM) estimates parameters of the model through the EM algorithm in dimensions defined by selected PCs. Briefly, the EM randomly initiates parameters ${\rm{\Theta }}$ (i.e. mean and variance of each cluster) then iteratively adjusts these parameters until convergence. By default, iterations stop when log-likelihood gain of the model is less than 1e−3.

For each observation, the expectation step (*E*) calculates likelihood of the observation belonging to clusters given current model parameters while the maximisation step (*M*) adjusts these parameters to maximise the likelihood of data. Once the model is trained, posterior probability of the gene to fall into each cluster is calculated. Finally, the gene is assigned to a cluster with the highest posterior probability.


$$\begin{equation*}{Q}_i = argma{x}_kP({Z}_i = k|{X}_i,{\rm{\Theta }})\end{equation*}$$

where ${Z}_i$ is a latent variable for *i*-th gene being in a *k*-th cluster and ${Q}_i$ is a cluster where *i*th gene is finally assigned.

#### Functional annotation of gene clusters

To test functional relevance of gene clustering, protein-protein interaction data as well as GO enrichment analysis using STRING data were performed (64). Gene clusters with significant PPI (FDR < 0.001) were identified and enriched GO terms were extracted.

#### STRING PPI analysis

To analyse functional connectivity between genes, STRING database was utilised. PPI enrichment was assessed with default settings which include both functional and physical protein associations. Network plots represent both a series of experimental evidence (i.e. protein fusion, neighbourhood, co-occurrence, co-expression and other experimental evidence) and computational predictions (i.e. text mining and database). It is recommended that PPI is used to validate the gene cluster as weak connectivity may indicate less biologically meaningful grouping.

#### Comparing expression and H3K27me3 breadth values to predict genes with different biological processes

Logistic regression models were trained independently with principal components of either expression or H3K27me3 breadth values pertaining to genes with different GO terms. A separate model was trained for each GO term, aiming to predict whether genes are associated with a particular term. Maximum values of breadth and expression were set to 1e5. The SMOTE algorithm (65) was used to oversample the under-represented class (i.e. genes with a given GO term).

#### Data visualisation of integrated data in a circular plot

We used the *circlize_dendrogram* from the *circlize* R package to create a circular dendrogram representing Jaccard similarities. The resulting dendrogram was then used to order the circular plot generated by the *circos-heatmap* function for the TRIAGE-Cluster peak number, and the proportion of cells in TRIAGE-Cluster peaks, based on original annotation and timepoints. The circular plots were compiled in Adobe Illustrator for visualization.

## RESULTS

**Figure 1.**
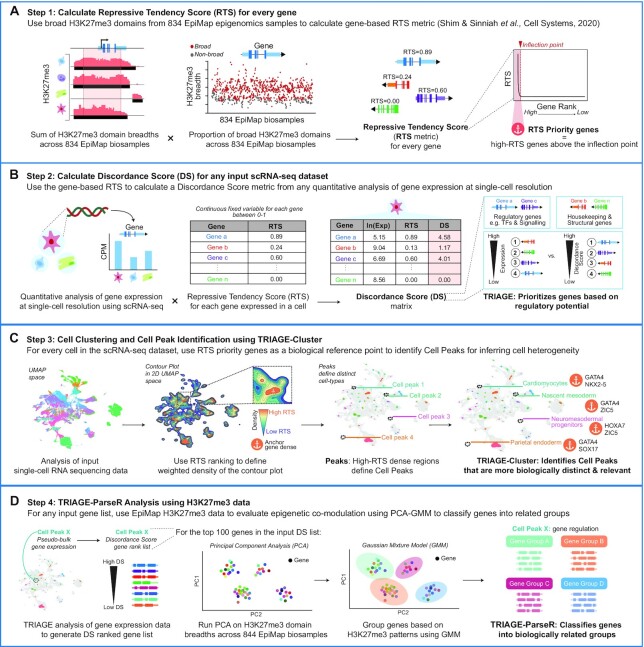
Overview of unsupervised pipeline for analysis of scRNA-seq data to identify cell types. (**A**) TRIAGE calculates a repressive tendency score (RTS) for every gene based on its association with broad H3K27me3 domains across 834 EpiMap bio-samples (59). RTS genes above the inflection point of the RTS curve are defined as RTS priority genes to assist in peak identification using TRIAGE-Cluster. (**B**) Input single cell expression matrix is transformed to discordance matrix to convert the original expression value to discordance score (DS). The DS results in high ranking of cell type regulatory genes. (**C**) We use RTS priority genes in a density-based clustering method, TRIAGE-Cluster, to identify cell populations in UMAP space. (**D**) For each peak, genes are ranked by pseudo-bulk discordance score (DS). TRIAGE-ParseR analysis uses PCA and Gaussian mixture model (GMM) to group genes into functional groups to assist with cell type identification.

Figure 1 provides a stepwise schematic for the workflow developed in this study, in which TRIAGE is used to determine cell populations and regulatory gene programs in scRNA-seq data. A glossary of terms is also provided in Table 1. First, TRIAGE determines the frequency of broad H3K27me3-domains at each protein coding gene locus across 834 EpiMap biosamples, resulting in a gene's repressive tendency score (RTS) (Supplementary Figure S1). Second, any orthogonal input gene expression data set (or any data mapped to protein coding genes) is multiplied with the RTS to derive a DS. The DS prioritizes genes with high regulatory potential (ranked by DS value). Third, TRIAGE-Cluster uses the top-ranked gene's RTS value for each cell as a weight to identify cell clusters in a single-cell UMAP space. Fourth, we use epigenetic data in a machine learning model to parse pseudo-bulk DS gene lists into biologically functional groups.

**Table 1.** Glossary of terms

RTS	Repressive tendency score. Calculated from broad H3K27me3 domains across 834 cell types. High RTS genes are lowly expressed and have higher cell-type-specific regulatory potential
Discordance score	Discordance score is calculated by multiplying log-transformed normalised gene expression values of any input data set with TRIAGE-derived RTS values for each gene
RTS Priority genes	Genes with RTS above the inflection point of the interpolated RTS curve are used to identify peak regions in the TRIAGE clustering method

### Using EpiMap data to calculate genome-wide repressive tendency scores

**Figure 2.**
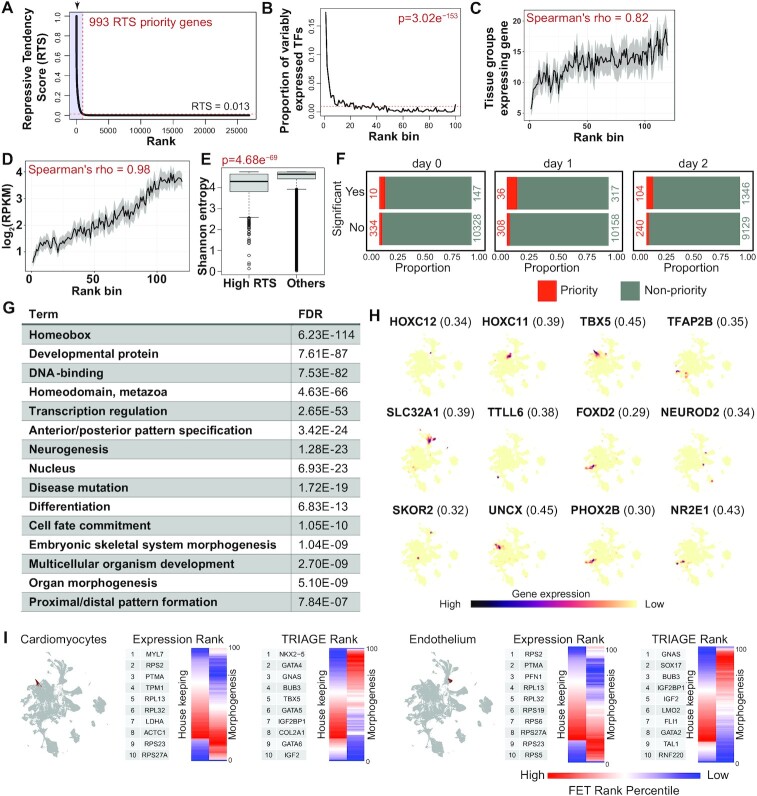
TRIAGE is a gene quantification and selection strategy providing a biological reference point for cell-specific genetic features. (**A**) 993 genes (RTS priority genes) have RTS values above the inflection point of the interpolated curve (red dashed line shows RTS = 0.013). (**B**) High-RTS genes are significantly associated with variably expressed transcription factors (VETFs). One-tailed Fisher's exact test shows the top 5%, representing 1340 RTS genes enriched with VETFs (*P* = 3.023e^−153^). Red dashed line represents the uniform distribution (proportion = 0.01). VETFs comprise 634 transcription factors (TFs) with variable expression across 46 Roadmap cell types (20,79). (C-E) Genes with high-RTS are associated with cell/tissue specificity (**C**), are lowly expressed (**D**) and have higher entropy (**E**). (**F**) Odds Ratio (OR) analysis demonstrating that RTS priority genes significantly influence the pseudotime trajectory of cell differentiation based on analysis of single-cell sequencing data characterizing definitive endoderm differentiation from 125 iPSC cell lines (38). The actual number of genes for each section are listed inside each plot. (**G**) Gene Ontology terms enriched in the top 993 RTS priority genes are significantly enriched in gene programs controlling cell identity, differentiation, and development. (**H**) UMAP plots of mouse gastrulation atlas data (41) showing selected RTS priority genes in the UMAP space indicative of cell-specific expression. RTS priority genes and their respective RTS are shown in parentheses. Mouse gene names were converted to human gene names for TRIAGE transformation. (**I**) TRIAGE transformation of gene expression re-ranks genes to enrich for genes with regulatory potential. This is shown for pseudo-bulk gene expression analysis of annotated cardiomyocytes (left) and endothelial cells (right) from mouse gastrulation atlas data (41). Data show UMAP of annotated cell types (left) followed by comparison of original expression vs TRIAGE gene ranking using top 10 ranked genes. The heatmaps showing distribution of housekeeping and morphogenesis genes ranked by original expression and TRIAGE. Enrichment is calculated for each rank bin relative to all genes (one-tailed Fisher's exact test).

Our first study (20) developed RTS using 111 NIH Epigenome Roadmap H3K27me3 samples and evaluated the consistency of RTS across various sample types. We demonstrate that RTS remain highly consistent regardless of the input samples cellular origins. In this study, we rank the expanded RTS generated across 834 cell samples and 27 tissue groups in EpiMap (20,59) (Table S2) and identify 993 priority genes above the inflection point of the interpolated RTS curve (RTS > 0.013) (Figure 2A). Highly variably expressed transcription factors (TFs) represent a positive gene set of cell type specific regulatory genes (20) and are significantly enriched among RTS priority genes (Figure 2B). Furthermore, genes ranked highly by RTS tend to be more cell type specific and lowly expressed (Figure 2C–E), a profile typical of regulatory factors, like TFs.

We evaluated whether the expression level of RTS priority genes influences cell differentiation more than would be expected by random chance. To test this, we analysed expression of RTS prioritised genes to evaluate their expression abundance correlated with differentiation trajectory using pseudotime analysis of definitive endoderm over three days of differentiation measured from 125 iPSC lines (38). These data show that expression of RTS priority genes between day 0–2 significantly affects the differentiation potential of cells (Odds Ratio (OR) on day 0 = 2.10, *P* = 0.04, day 1 = 3.75, *P* = 7 × 10^−14^, and day 2 = 2.94, *P* = 2.7 × 10^−20^) (Figure 2F). This is borne out by data showing that high-RTS genes are enriched in DNA-binding factors such as homeobox genes (FDR 6.2e^−114^) controlling fundamental processes in cell differentiation, development, and tissue morphogenesis (Figure 2G).

We next tested whether the RTS values could be used as an orthogonal metric to identify cell types in scRNA-seq data. We evaluated data sets for method development and selected a mouse gastrulation atlas data which profiles 116 312 cells collected at nine sequential time-points ranging from e6.5 to e8.5 days post-fertilisation (41) (Supplementary Figure S2 and S3). These data comprise all primary germ layers, consisting of 37 classified cell types including ectoderm, mesoderm, and endoderm (Supplementary Figure S2). We first find that high-RTS ranked genes have cell-specific expression patterns across the UMAP space (Figure 2H). Second, analysis of pseudo-bulk gene expression of two annotated cell types, cardiomyocytes and endothelial cells shows that original gene expression enriches for housekeeping and functional genes, while the TRIAGE transformation to DS enriches for regulatory genes underpinning the mechanistic basis of heart (*NKX2-5*, *GATA4*, *TBX5*) and endothelial (*SOX17*, *GATA2*, *TAL1*, *LMO2*) development and morphogenesis (Figure 2I).

### TRIAGE RTS demarcates cell specific clusters in scRNA-seq data

**Figure 3.**
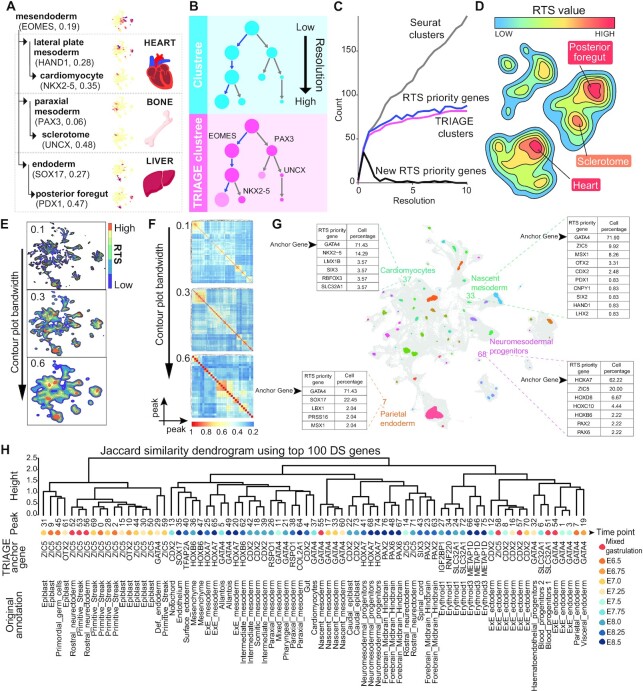
TRIAGE-Cluster is an unsupervised approach to enable cell type identification in a UMAP space. (A, B) Example (**A**) and schematic (**B**) demonstrating how RTS priority genes provide a quantitative value assigned to all genes which can be exploited for cell-type identification. (A) The quantitative value of RTS priority genes enables identification of specific cell types where increasing RTS values correlate with increasing cell type specificity. UMAPs in the middle show expression of selected priority genes. (B) Strategy for implementing decision making criteria when splitting clusters from low to high resolution in normal clustering and TRIAGE-Cluster. (**C**) Evaluation of TRIAGE decision-making criteria using Seurat clustering resolutions. Seurat cluster: number of clusters at each Seurat resolution. RTS priority genes: cumulative number of RTS priority genes across each Seurat resolution. TRIAGE clusters: number of clusters with at least one RTS priority gene expressed at each Seurat resolution. New RTS priority gene: number of new RTS priority genes at each resolution. (**D**) Schematic concept for using RTS priority genes as biological anchoring approach to analyse scRNA-seq data sets. We use a weighted density analysis of RTS priority genes in each cell to derive a topographic landscape view of the data with defined cell types identified as peaks in the contour plot. (**E**) Contour plot analysis applied to single cell atlas of mouse gastrulation atlas data (41) with granularity from high to low in visualisation using parameter bandwidth setting. (**F**) Heatmaps with bandwidth resolutions (E) showing pairwise Jaccard similarity score between peaks calculated from gene discordance score for each cluster. (**G**) UMAP of single cell atlas of mouse gastrulation atlas data using TRIAGE-Cluster with bandwidth setting of 0.3. Four examples show the RTS priority genes (anchor genes) expressed in the majority of cells within the peak and annotation assigned by the original classification (41). (**H**) Jaccard similarity dendrogram with bandwidth 0.3 (F) showing peak relationship (first row), mapped timepoint (second row), anchor gene (third row), and mapped original annotation (forth row).

Figure 3A demonstrates a model for how RTS assigned to genes by TRIAGE quantifies a distinction between non-specific vs cell type-specific genes. For example, *EOMES* represents a broad mesendoderm cell type (RTS 0.19), while more specific markers of lateral plate mesoderm (*HAND1*, RTS: 0.28) and cardiomyocytes (*NKX2-5*, RTS: 0.35) demonstrate that RTS values can be used as a surrogate indicator for genes demarcating biologically unique cell types. Similar examples are shown for paraxial mesoderm and endoderm (Figure 3A). To test this model, we analysed time-course data of cell differentiation (39) and evaluated RTS scores in cell types from early mesendoderm into differentiated cell types (Supplementary Figure S4a). These data demonstrate that RTS scores do not simply increase with differentiation into definitive cell types. Instead, high RTS genes can be used to identify biologically distinct cell types at every stage of differentiation irrespective of development stage or lineage (Supplementary Figure S4b). Next, we aimed to test this hypothesis by developing a single cell clustering method that utilizes RTS values for cell type identification.

We first tested whether RTS assigned to genes could provide a method to identify gene clusters in scRNA-seq data. We designed a proof-of-concept analysis comparing Seurat clustering versus RTS priority genes as a decision-making feature influencing clustering tree analysis over 10 resolutions (Figure 3B). Standard Seurat clustering progressively divides clusters with increasing resolution (Figure 3C). For TRIAGE-Cluster, we assumed enrichment of RTS priority genes in a cell population demarcates unique cell types and evaluated this at each stepwise resolution of clustering. We found that RTS priority genes characterising new clusters quickly plateaus demonstrating that increased clustering resolutions do not necessarily imply biologically distinct cell types. Furthermore, RTS priority genes trimmed the number of clusters, while still being sensitive enough to identify new clusters even at very high resolutions, representing rare cell types that cannot be detected in broad categorizations at low resolutions (Figure 3C).

### Weighted density analysis of RTS priority genes determines cell clustering

Based on these observations, we developed an approach drawing on the concept of a topographic map with contour lines indicating peaks of cell specificity in a low-dimensional gene expression profile (Figure 3D). We hypothesised that RTS prioritised genes expressed in cells demarcate cell-specific identity and a gene's RTS can define contour lines that separate peaks (specific cell types) from valleys (transitional or non-specific cell types) (Figure 3D). Therefore, we treated RTS as the weighting parameter to adjust and estimate cell density in a low-dimensional whole transcriptome space. We selected 2D UMAP embeddings instead of t-SNE, as UMAP better preserves the global structure of scRNA-seq data (44) which is supported by a comparative analysis of t-SNE and UMAP methods (Supplementary Figure S5a, b).

We applied this method to the mouse gastrulation atlas data (41) (Figure 3E–H). Bandwidth resolutions are shown for the contour plot (Figure 3E) and pairwise Jaccard similarity between peaks (Figure 3F). Figure 3G shows the cell clustering result at bandwidth 0.3 identifies 77 peaks. These peaks demonstrate that TRIAGE-Cluster is capable of capturing the cell diversity of the data, as all annotations in the original data are identified in our clustered cell types (Supplementary Table S3). A further confirmation of this using a second data set of human adult heart with myocardial infarction (66) shows consistent results that all annotations from the original data are retained and represented with one or more peaks, as determined by TRIAGE-Cluster (Supplementary Table S4). For selected populations we show how high-RTS genes, which we call anchor genes, reveal identity-defining genes across different cell types in the data. For example, cardiomyocytes are anchored primarily by the mes-endoderm-associated gene *GATA4* and cardiac regulatory factor *NKX2-5*. Parietal endoderm is anchored by *GATA4* and the endoderm-associated transcription factor *SOX17*.

We next evaluated peak-to-peak associations obtained through the pairwise Jaccard similarity analysis of the op 100 DS genes in each peak of the mouse gastrulation atlas data (Figure 3F). The output provides an integrated view of TRIAGE-Cluster derived peak relationships in which we show (i) the output dendrogram organising peak relationships using DS, (ii) each peak's time point as it appears during embryonic development, (iii) the original annotation for each peak from the mouse gastrulation atlas data and (iv) the highest expressing RTS priority gene which we call the peak's ‘anchor gene’ (Figure 3H and Figure S6).

### TRIAGE-cluster helps capture granular cell diversity in scRNA-seq data

**Figure 4.**
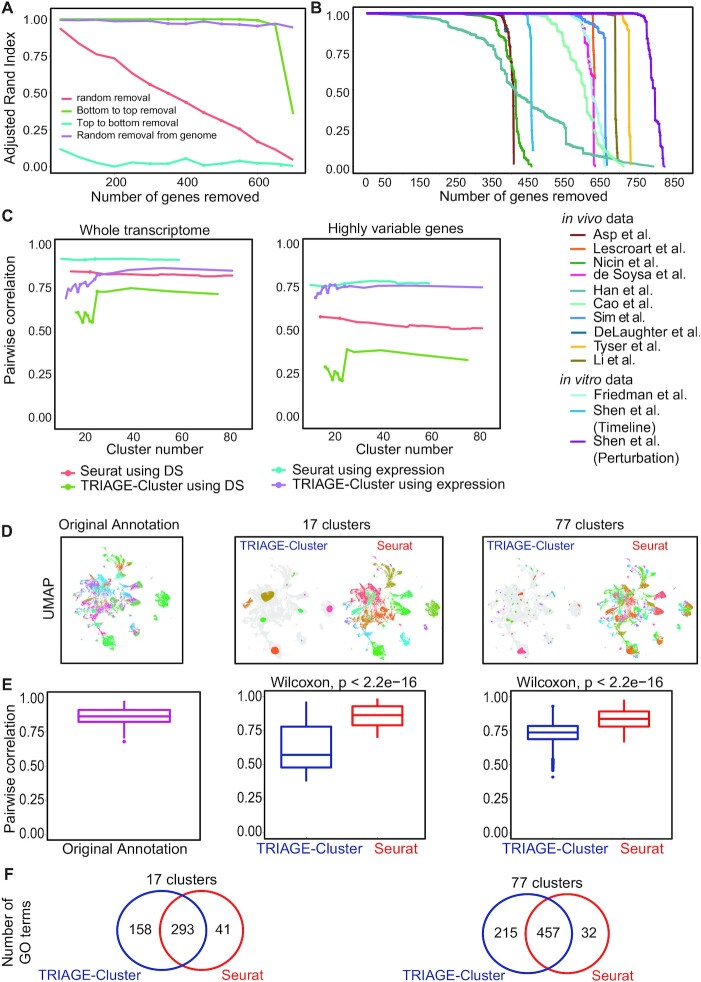
TRIAGE-Cluster identifies biologically distinct cell types. (**A**) Adjusted Rand Index analysis of single cell atlas of mouse gastrulation atlas data (41) demonstrating that the quantitative RTS priority gene rank is required for accurate cell clustering. (**B**) Thirteen data sets from *in vivo* and *in vitro* cell atlases analysed for clustering performance by removal of RTS priority genes from bottom to top demonstrate a critical role for high-RTS genes in clustering accuracy. (**C**) Comparison of peak-peak similarity using Spearman rank correlation of whole transcriptome (left) and highly variable genes (right). We evaluate the correlation with the mouse gastrulation atlas data (41) of original expression and TRIAGE transformed matrix (DS matrix), showing Seurat and TRIAGE-Cluster with increased number of clusters at 20 resolution/bandwidths (0.1–2 with 0.1 increments). (**D**) UMAPs of the mouse gastrulation atlas data showing TRIAGE-Cluster and Seurat peaks. Quantitative analysis demonstrates peak-peak correlation comparing original annotation cell diversity compared to TRIAGE-Cluster and Seurat when cell diversity is fixed at 17 or 77 clusters. (E, F) Spearman rank correlation (**E**) and Gene Ontology analysis (**F**) demonstrate that TRIAGE-Cluster gives significantly lower correlation and therefore captures greater differences in cell diversity (E) and supported by increased gene ontology enrichment compared to Seurat or original annotations (F).

We next analysed the sensitivity of TRIAGE-Cluster to parameter settings that help determine the clusters. We first asked whether removal of RTS priority genes influences the clustering output using an Adjusted Rand Index (ARI) analysis. While random removal of genes from the genome has no impact on TRIAGE-Cluster accuracy, it is not unexpected that the most dramatic impact on clustering accuracy occurs with removal of high-RTS genes (Figure 4A). This is further demonstrated across thirteen independent *in vivo* and *in vitro* scRNA-seq data sets (39,45,63,67–75) (Supplementary Table S5) which shows that high-RTS genes are the primary drivers of clustering across all data sets (Figure 4B). We assessed this further to address whether RTS-prioritised genes are under-represented in sparse scRNA-seq data. Despite the typically low expression levels of high-RTS genes in scRNA-seq dataset, our comparison of the original and MAGIC-imputed (57) versions of mouse gastrulation atlas data reveals that the peaks identified in the original data capture most of the high-RTS genes and exhibit greater biological diversity compared to MAGIC-imputed data (Supplementary Figure S5c, d).

We next tested the performance of TRIAGE-Cluster using the assumption that distinct cell types have gene expression differences reflected by lower cluster-cluster pairwise correlation. Comparing TRIAGE-Cluster against Seurat, we tested gene expression similarity between all pairwise peaks at different clustering resolutions using Spearman rank correlation. Seurat clustering shows highly stable similarity between cell types at all clustering resolutions regardless of input data type (original expression or DS, i.e. when expression is multiplied by RTS). Furthermore, Seurat shows stable correlation between clusters using either the whole transcriptome or highly variable genes for gene expression similarity comparison. This suggests that separation of distinct cell types over different cluster resolutions in Seurat is not driven by significant global gene expression differences (Figure 4C). To further test the performance of TRIAGE-Cluster, we compared the peaks identified by TRIAGE-Cluster with the clusters identified by Seurat and SC3. By using ARI across different cluster numbers and at 77 peaks/clusters, we found that TRIAGE-Cluster outperformed the other methods (Supplementary Figure S5e, f).

In contrast, TRIAGE-Cluster peaks consistently result in lower peak-peak Spearman rank correlation compared to Seurat and the correlation increases at higher clustering resolutions as differences between cell types become less distinct (Figure 4C). We achieved the lowest peak-peak correlation using TRIAGE-Cluster with highly variable DS values assigned to genes. We further demonstrate this comparing peak-peak correlations of TRIAGE-Cluster versus Seurat clusters in which the clustering resolution of both methods results in the same cluster number (Figure 4D). Peak-peak correlations of cell types originally defined in the single cell atlas of mouse gastrulation atlas data (41) were similar to those defined by Seurat (Figure 4D-E). To further validate that TRIAGE-Cluster peaks capture functionally relevant gene sets in the data, we performed Gene Ontology analysis using the genes with the top 100 DS for each peak or cluster. These data show that TRIAGE-Cluster peaks have more GO BP terms than Seurat clusters and therefore demonstrates that TRIAGE-Cluster extracts genes involved in more diverse biological processes (Figure 4F, Tables S6–S9). To evaluate alternate clustering methods we compared TRIAGE-Cluster and SC3 (56). While Seurat uses a graph-based approach to connect cells based on their mutual nearest neighbour relationships, SC3 constructs a consensus matrix to represent the similarity between cells based on clustering results from multiple algorithms. As with comparison to Seurat, we found that peaks generated by TRIAGE-Cluster exhibit greater differences in cell diversity than those from SC3 based on enrichment in gene ontologies represented in the clusters (Supplementary Figure S5g–j). Taken together, these data show that a combination of DS score with TRIAGE-Cluster efficiently identifies biologically distinct cell types in scRNA-seq data.

In our companion study, we demonstrate the utility of TRIAGE-Cluster by applying it on a multiplexed single cell atlas of iPSC differentiation data that integrates temporal data across eight time points and signalling data with nine developmental signalling perturbation (71). Forty-eight peaks were identified from the *in vitro* differentiation atlas data, and clustering performance was evaluated using a trajectory approach VIA, which evaluates cell states’ relationship (76). Comparing analysis using all cells vs TRIAGE-Cluster peaks, TRIAGE-Cluster peaks give better peak-to-node assignment in the trajectory and significant correlation between pseudotime and actual time. These data demonstrate that TRIAGE-Cluster can define the biological diversity and improve cell-cell trajectory analysis in scRNA-seq data. Overall, the assessment of the impact of RTS gene on clustering output using thirteen independent scRNA-seq datasets (Figure 4A, Table S5) presented in this manuscript, along with our companion study applying the TRIAGE-Cluster pipeline to iPSC differentiation data covering diverse lineages from *in vitro* and *in vivo* development, provide compelling evidence for the generalizability of our method across different assay conditions such as drug perturbations.

### TRIAGE-ParseR enhances identification of cell gene regulatory networks

To complement TRIAGE-Cluster, we developed a method called TRIAGE-ParseR to extract regulatory information from a distinct population of cells. We aimed to develop a method that distinguishes biologically meaningful gene groups without reliance on subjective external reference points (such as differential expression) or prior knowledge (such as GO, STRING, marker gene selection, or gene class biases) (19).

Based on the rationale that expression of key developmental genes is modulated by both gain or loss of H3K27me3 (77,78), we hypothesized that principal component analysis (PCA) of (breadth of) H3K27me3 for genes across diverse cell states, specifically in diverse human tissue and cell types, could help separate functionally relevant gene-gene relationships. By training logistic regression models with principal components obtained from gene expression or H3K27me3 breadth values, we demonstrate superiority of H3K27me3 as the input feature to predict genes with various Gene Ontology terms (Supplementary Figure S7)

**Figure 5.**
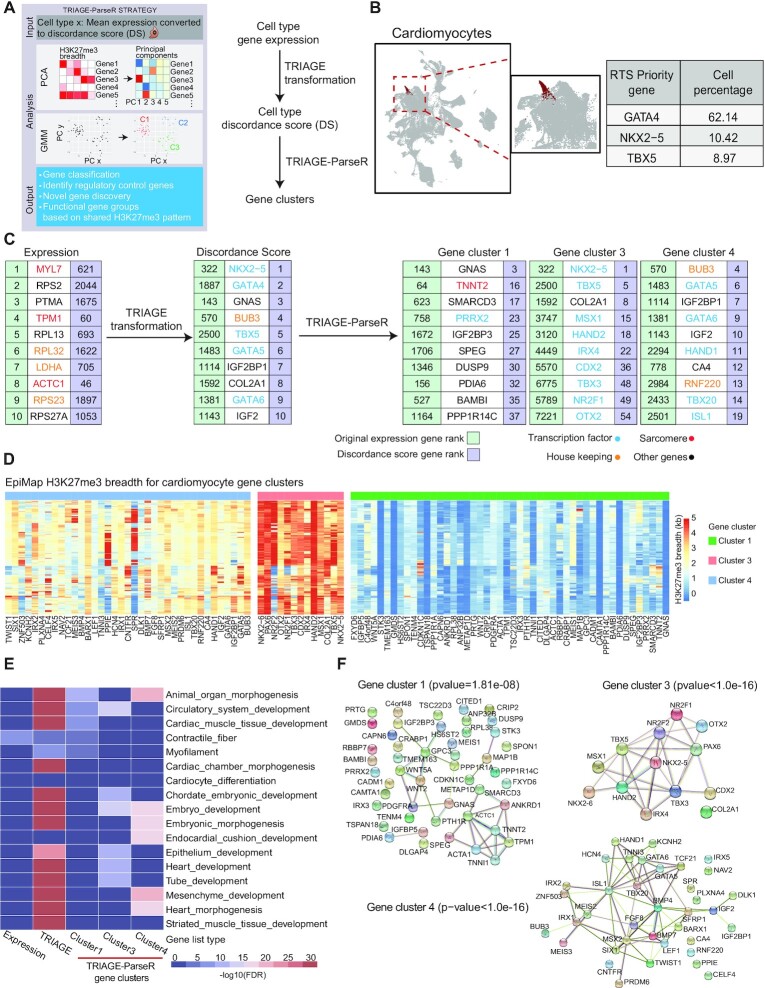
TRIAGE-ParseR clusters genes into groups using unsupervised analysis of epigenetic data. (**A**) Schematic workflow describing TRIAGE-ParseR which uses H3K27me3 across hundreds of bio-samples to cluster genes using GMM on PCA space to improve analysis of cell gene regulatory networks. (**B**) UMAP of cardiomyocytes from mouse gastrulation atlas data showing top 3 RTS priority genes expressed in cardiomyocytes. (**C**) Top 10 genes in annotated cardiomyocytes ranked by original expression (left), discordance score (middle), and top 10 genes in gene clusters resulting from TRIAGE-ParseR. (**D**) Abundance of H3K27me3 deposited across each gene locus measured from EpiMap bio-samples demonstrate unique epigenetic patterns demarcating specific gene groups shown in panel C. (**E**) Heatmap showing gene ontology enrichment (−log_10_ (FDR)) across top 100 ranked genes from cardiomyocytes ranked by original expression (first column), discordance score (second column), and TRIAGE-ParseR gene groups (last three columns). (**F**) String gene network analysis showing protein-protein interactions of the three cardiomyocyte gene clusters.

After performing PCA, we evaluated the input genes list for shared H3K27me3 deposition patterns encoded by the top PCs and modelled by a GMM. To generate the input gene list, the original expression matrix of a cell type is converted to DS and evaluated using TRIAGE-ParseR (Figure 5A). Extracted H3K27me3 patterns by PCA showed that (i) most of the data variance can be explained by a small number of PCs (50) (Supplementary Figure S8a), (ii) genes enriched with a specific H3K27me3 pattern demonstrate tissue-specific biological processes (Supplementary Figure S8b), and (iii) distinct tissue types can be characterised by these H3K27me3 patterns (Supplementary Figure S8c). Furthermore, these data show that the first PC recovers genes with strong tendency to deposit broad H3K27me3 across many tissue types (i.e. high-RTS genes) while subsequent PCs capture more tissue type-specific patterns (Supplementary Figure S8d). This justifies the importance of incorporating diverse PCs to reveal biologically informative signals that distinguish gene programs underpinning different cell types. We used TRIAGE-ParseR to evaluate gene expression from heart tissue and blood (Supplementary Figure S8e–g). In addition to the first PC which shows high concordance with RTS, analysis of diverse PCs reveals unique gene-gene TRIAGE-ParseR signatures that can be used to inform gene-gene relationships.

We applied TRIAGE-ParseR on single cell clusters identified in the mouse gastrulation atlas data and focussed the initial demonstration of the method on cardiomyocytes (Figure 5B). In the workflow pipeline (Figure 5C), pseudo-bulk gene expression (top ranked are primarily housekeeping and structural genes) is transformed with TRIAGE to derive the DS (top ranked are primarily cardiac regulatory genes). The top 100 genes ranked by DS are used as input to TRIAGE-ParseR as this gene number gave strong enrichment of variably expressed TFs as the surrogate of developmentally regulated genes (79) (Figure 5C, Figure S8h). The result showed three gene clusters with significant enrichment by STRING analysis (protein–protein interaction FDR < 0.001). Figure 5D shows the raw H3K27me3 deposition patterns that clearly demarcate common epigenetic patterns governing each gene group.

We compared GO enrichment at each step of the workflow (Figure 5C). While original gene expression shows enrichment in terms related to structural features of cardiomyocytes, TRIAGE DS provides robust enrichment in diverse pathways associated with cardiomyocyte morphogenesis and differentiation, TRIAGE-ParseR separates these genes into groups. Genes in cluster 3 (the most significantly repressed genes across diverse cell types, Figure 5D) are enriched with heart development terms including diverse transcription factors known to be critical for heart development (Figure 5E). Clusters 4 genes have intermediate repression (Figure 5D) and are enriched in generic genes governing embryo development (Figure 5E). Lastly, genes in cluster 1 have minimal repression across diverse cell types (Figure 5D) and are enriched in genes involved in cardiac muscle function (Figure 5E). Indeed, STRING analysis revealed that genes in these clusters formed significant protein-protein interactions and therefore revealed both known and unknown gene-gene relationships (PPIs, enrichment *P* < 0.001) (Figure 5F). These data demonstrate that H3K27me3 patterns reveal gene regulatory networks relevant to biological functions of genes and TRIAGE-ParseR can meaningfully segregate genes to facilitate cell type identification and biological interpretation.

### Combing TRIAGE-cluster and TRIAGE-ParseR to identify cell types in scRNA-seq data

**Figure 6.**
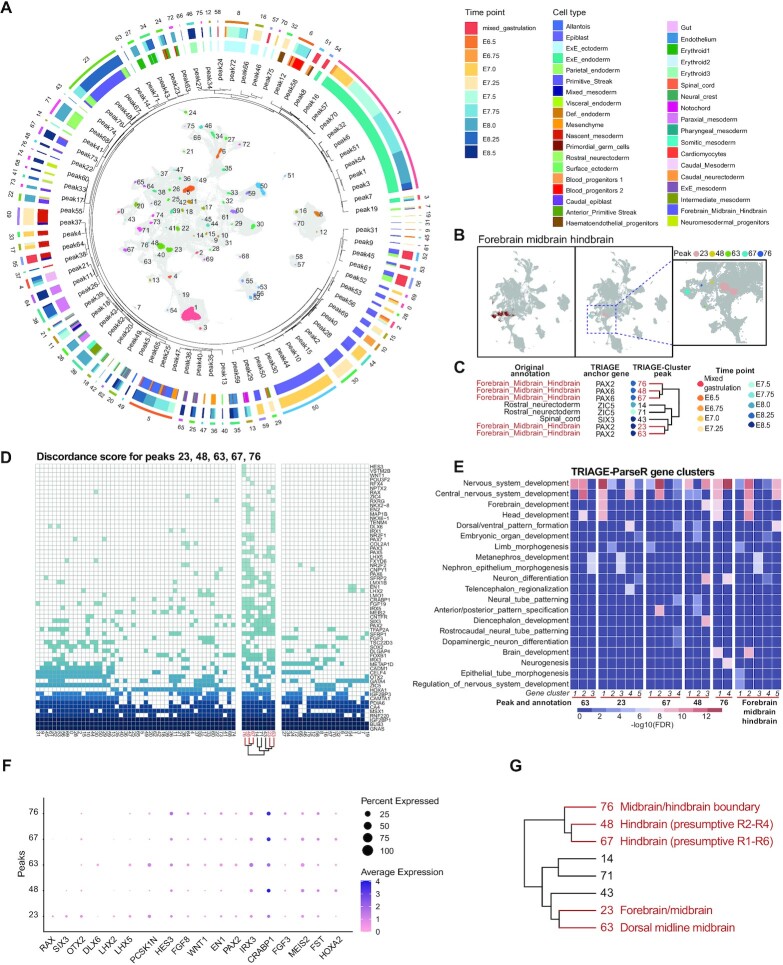
TRIAGE-Cluster enables efficient and unsupervised identification of cell subtype diversity in scRNA-seq data. (**A**) Integrated metadata for all peaks identified by TRIAGE-Cluster analysis in the mouse gastrulation atlas data. From inside to out: data include UMAP identifying TRIAGE-Cluster peaks, peak relationships based on 0.3 bandwidth dendrogram (Figure 3H), TRIAGE-Cluster peak number, proportion of cells in TRIAGE-Cluster peaks based on original annotation and timepoints. (**B**) UMAP of original annotation of forebrain midbrain hindbrain (left) and location of TRIAGE-Cluster peaks (right and inset). (**C**) Jaccard similarity dendrogram with bandwidth 0.3 showing five distinct ‘forebrain midbrain hindbrain’ TRIAGE-Cluster peaks with their respective mapped timepoint and anchor genes (extracted from Figure 2H). (**D**) Specificity of discordance score genes identified for the five ‘forebrain midbrain hindbrain’ peaks compared to all TRIAGE-Cluster peaks. A coloured box indicates a gene (row) characterises that peak (column). Peaks sharing a characteristic gene indicates shared regulatory or structural features defining that cell type. (**E**) Heatmap showing different neural cell subtype gene ontology enrichment (–log_10_ (FDR)) from TRIAGE-ParseR across gene clusters in the five forebrain midbrain hindbrain peaks (the first five blocks) and original forebrain midbrain hindbrain cells (the last block). (**F**) Dot plot showing marker genes associated with neural lineage subtypes in each forebrain midbrain hindbrain peak. (**G**) Jaccard similarity dendrogram showing revised cell group classifications for the original forebrain_midbrain_hindbrain cell type using combined analysis of TRIAGE-Cluster and TRIAGE-ParseR.

We utilised both TRIAGE-Cluster and TRIAGE-ParseR as a collective suite of methods to evaluate cell types in the mouse gastrulation atlas data. Figure 6A shows a holistic view of all TRIAGE-Cluster peaks, peak relationship at bandwidth 0.3, as well as corresponding original annotation and timepoint. While the mouse gastrulation atlas data set provides a powerful resource for studying the earliest stages of mammalian organogenesis, most cell classifications capture redundant or broad cell groups that lack the nuance of cell diversification during early development. We therefore implement TRIAGE-Cluster as a complementary strategy for identifying more distinct cell types based on its ability to determine local maxima of RTS-ranked genes in the data set as a surrogate indicator of biologically distinct cell populations.

We selected cells annotated as forebrain/midbrain/ hindbrain in the original data. TRIAGE analysis identifies five peaks (23, 48, 63, 67, and 76) mapped to this annotation (Figure 6B). We provide a multi-scale data output to evaluate identity-defining features of each cell type including their relationships defined by TRIAGE-derived dendrogram (Figure 6C), the highest ranked DS genes for each cell type (Figure 6D), and TRIAGE-ParseR analysis of gene–gene groupings analysed by GO analysis (Figure 6E). These data reveal a separation of distinct cell relationships within this broad cell grouping that suggest more nuanced cell subtypes governing ectoderm development. Clusters 48 and 67 share high degrees of similarity based on their gene ontology enrichment for neural patterning (Neural tube, Dorsal/Ventral and Anterior/posterior pattern specification, Figure 6E) and DS with genes including the shared TRIAGE anchor gene *PAX6*, an organizer of hindbrain patterning. Detailed gene expression data suggest cluster 67 is boundary-defining cells of presumptive rhombomeres R1-R6 (*CRABP1* and DS for *FGF3*) while cluster 48 is more specifically associated with presumptive R2-R4 (*FST* and *HOXA2*, Figure 6F). Cluster 76, anchored by *PAX2*, shares a broad range of identity defining genes related to establishment of the Isthmic organizer at the midbrain/hindbrain boundary (*HES3*, *FGF8*, *WNT1*, *EN1*). In contrast, clusters 23 and 63 share similarity based on the TRIAGE-dendrogram (Figure 6C). Cluster 23 shows signatures of Forebrain/midbrain marked by *RAX*, *SIX3*, *LHX2*, *LHX5* and *PCSK1N* (Figure 6F) and GO enrichment for Forebrain development (Figure 6E). Cluster 63 is associated with the dorsal midline of the midbrain marked by *PCSK1N*, *HES3*, *FGF8* and *DLX6*. We provide a second analysis of paraxial mesoderm lineage cell types based on detailed dissection using TRIAGE-associated outputs (Supplementary Figure S9). Further examples of the data interpretation pipeline outlined here are provided for analysis of *in vitro* differentiation from pluripotency outlined in the companion paper (71). The collective results of both studies including new data, software package downloads, and data analysis dashboard can be accessed online (http://cellfateexplorer.d24h.hk/).

## DISCUSSION

Understanding biological relationships between cell types and the gene networks that govern these differences remains central to maximising the value of technologies capturing genomic data at single cell resolution. This study demonstrates that tools like TRIAGE that use an orthogonal reference point to determine identity-defining features provide a powerful mechanism for defining and interpreting the genetic basis of cell populations. The values assigned to all genes based on their RTS can act as an independent biological reference point, which can be implemented downstream of dimensionality reduction algorithms to cluster cell types.

Regulation of the genome is in part mediated through histone modifications that control genome accessibility, acting as an on/off switch to regulate gene programs (80). We reasoned that regions with the broadest histone modification domains (representing extreme levels of on/off control) could be used to quantify the probability that a locus has a role controlling cell decisions and functions. Based on this rationale, TRIAGE (20) uses patterns of histone methylation data to identify regions of the genome that are enriched in cell-specific identity defining genes. Genes with a high RTS have consistent repressive chromatin in most cell types and, therefore, in the rare instances when these genes get turned on, they likely have key roles controlling cell decisions or functions.

The collective suite of methods built around TRIAGE provide several major advantages: (i) TRIAGE is a customisable metric with a single value assigned to genes which makes it simple to implement in any genomic data set, (ii) it is versatile, providing a quantitative genomic model to weight gene prioritisation using routine genetic analysis tools, (iii) the simplicity of TRIAGE enables broad implementation in data analysis pipelines for any data sample from any cell, tissue, disease, or individual and providing a method to enrich for factors most likely to cause phenotypic changes in cell identity and (iv) can help prioritise genetic targets for drug development and identifying the primary determinants of disease and development.

TRIAGE-Cluster incorporates gene-level information inferred from H3K27me3, which offers a restricted view of biologically relevant cell populations in scRNA-seq data and provides higher sensitivity for detecting rare cell types. Our evaluation of TRIAGE-Cluster on *in vitro* and *in vivo* scRNA-seq datasets shows that it can accurately identify at least one peak in every annotated cell type of the data (Supplementary Tables S3 and S4). This feature makes TRIAGE-Cluster highly suitable for generalizable use across a wide range of developmental and adult tissues, as well as various assay conditions. Since our analysis demonstrates peaks generated from TRIAGE-Cluster can capture greater differences in cell diversity compared to current methods (Figure 4 and Figure S5), we introduce it as a complementary method to baseline clustering methods to help identify transcriptionally distinct cell types and gene expression patterns that may be missed by other methods. TRIAGE-ParseR provides complementary perspectives in studying gene-gene relationships. Unlike ChromHMM (81) or Paige *et al.*’s work (82), which focus on exploring the combinatorial relationship between various histone modifications and other genomic features in a specific cellular context, TRIAGE-ParseR leverages a diverse range of biological contexts. This approach allows the tool to extract epigenetic patterns defined by H3K27me3 that are universally applicable. Namely, (i) it provides an unsupervised approach to study the gene regulatory basis of cells without drawing on external reference points (like differential expression analysis) or prior knowledge (including gene expression data), (ii) it parses genes into groups to reveal biological mechanisms underpinning cell states and (iii) uses an independent reference data set for evaluating epigenetic patterns which provides a mechanism for applying this method to any input gene list in various biological contexts.

In future studies, we aim to address three limitations in the current workflow. First, we demonstrate that the current clustering method significantly restricts cell selection to capture clusters with the most biologically distinct identity. Our method has demonstrated the ability to capture theoretically any cell type from diverse data types (Supplementary Tables S3 and S4), and furthermore, it has the potential to offer greater clarity and insight into the diversity of cell subtypes within a given population. While this is a major advantage, it comes at the cost of data trimming which should be weighed relative to the loss of statistical power provided by the scale of cell sampling. To address this limitation, we aim to use TRIAGE-Cluster in combination with data imputation or trajectory inference methods to interpret cell-cell relationships using all cells in the data set. Second, the current method is reliant on assumptions built into dimensionality reduction representing cell relationships in UMAP space. We indeed demonstrate that peaks generated from UMAP exhibit greater biological diversity compared with another topological dimensional reduction method, t-SNE (Supplementary Figure S5a, b), but the major limitations exist for simplifying the data into these graphical presentations. To address this, we propose additional strategies such as comparison of cell types using TRIAGE-dendrograms (Figure 3H, Figure S6) which provide independent approaches to evaluate cell-cell relationships. We aim to develop approaches that provide the analysis value of TRIAGE-Cluster but avoid the limitations associated with topographical map representations. Third, TRIAGE-ParseR provides a unique approach to decipher gene-gene relationships but draws on only a single epigenetic reference point. Future developments of this method need to parameter test additional histone modifications representing active promoters (H3K4me3) or enhancers (H3K27ac and H3K4me1) as complementary to repressive chromatin marked by H3K27me3 as a basis for more nuanced analysis of gene-gene relationships.

This study introduces a unique strategy for identifying cell types by using epigenetic information as a biological reference point for cell clustering and deconstructing gene modules in single-cell expression data. The workflow is illustrated by analysing *in vivo* organogenesis and *in vitro* differentiation atlases. Collectively, TRIAGE highly ranks regions of the genome enriched in genetic features with profound effects on cell identity because these regions encode potent determinants of cell developmental processes. These ranking features and genome-wide predictions make TRIAGE unique among genomic analysis methods and position it to address areas of need in cell biology and genetics. In addition to our prior studies (20), TRIAGE-Cluster and TRIAGE-ParseR provide a growing portfolio of methods that complement baseline methods such as Seurat and SC3. These methods facilitate analysis of large-scale data sets by integrating genome-wide epigenetic data with scRNA-seq data to parse cell-cell and gene-gene relationship governing mechanisms of cell identity.

## DATA AVAILABILITY

The mouse gastrulation atlas data is available in *MouseGastrulationData* (83) at https://github.com/MarioniLab/MouseGastrulationData. Code are available on Zenodo for TRIAGE-Cluster (https://doi.org/10.5281/zenodo.7816427) and TRIAGE-ParseR (https://doi.org/10.5281/zenodo.7816635). The combined analysis of the mouse gastrulation atlas data and *in vitro* iPSC differential data can be explored at http://cellfateexplorer.d24h.hk/. Software used in this study are listed in Supplementary Table S10.

## Supplementary Material

gkad307_Supplemental_Files

## References

[1] Shen S. , Sun Y., Matsumoto M., Shim W.J., Sinniah E., Wilson S.B., Werner T., Wu Z., Bradford S.T., Hudson J. et al. Integrating single-cell genomics pipelines to discover mechanisms of stem cell differentiation. Trends Mol. Med. 2021; 27:1135–1158. 34657800 10.1016/j.molmed.2021.09.006

[2] Kiselev V.Y. , Andrews T.S., Hemberg M. Challenges in unsupervised clustering of single-cell RNA-seq data. Nat. Rev. Genet. 2019; 20:273–282. 30617341 10.1038/s41576-018-0088-9

[3] Guo M. , Wang H., Potter S.S., Whitsett J.A., Xu Y. SINCERA: a Pipeline for Single-Cell RNA-Seq Profiling Analysis. PLoS Comput. Biol. 2015; 11:e1004575. 26600239 10.1371/journal.pcbi.1004575 PMC4658017

[4] žurauskiene J. , Yau C. pcaReduce: hierarchical clustering of single cell transcriptional profiles. BMC Bioinf. 2016; 17:140. 10.1186/s12859-016-0984-y PMC4802652 27005807

[5] Lin P. , Troup M., Ho J.W.K. CIDR: ultrafast and accurate clustering through imputation for single-cell RNA-seq data. Genome Biol. 2017; 18:59–59. 28351406 10.1186/s13059-017-1188-0 PMC5371246

[6] Parasa N.A. , Namgiri J.V., Mohanty S.N., Dash J.K. Data Analytics in Bioinformatics. 2021; 35–49.

[7] Costa I.G. , de Carvalho F.d.A.T., de Souto M.C.P. Comparative analysis of clustering methods for gene expression time course data. Genet. Mol. Biol. 2004; 27:623–631.

[8] Jelili O. , Itunuoluwa I., Funke O., Olufemi A., Efosa U., Faridah A., Moses A., Ezekiel A. Clustering algorithms: their application to gene expression data. Bioinform. Biol. Insights. 2016; 2016:237–253. 10.4137/BBI.S38316 PMC5135122 27932867

[9] Yu B. , Chen C., Qi R., Zheng R., Skillman-Lawrence P.J., Wang X., Ma A., Gu H. scGMAI: a Gaussian mixture model for clustering single-cell RNA-Seq data based on deep autoencoder. Brief. Bioinf. 2020; 22:bbaa316. 10.1093/bib/bbaa316 33300547

[10] Ester M. , Kriegel H.P., Sander J., Xiaowei X. A Density-based Algorithm for Discovering Clusters in Large Spatial Databases with Noise. 1996; Menlo Park, CA (United States) AAAI Press.

[11] Jiang L. , Chen H., Pinello L., Yuan G.-C. GiniClust: detecting rare cell types from single-cell gene expression data with Gini index. Genome Biol. 2016; 17:144. 27368803 10.1186/s13059-016-1010-4 PMC4930624

[12] Andrews T.S. , Hemberg M. Identifying cell populations with scRNASeq. Mol. Aspects Med. 2018; 59:114–122. 28712804 10.1016/j.mam.2017.07.002

[13] Wu H. , Mao D., Zhang Y., Chi Z., Stitzel M., Ouyang Z. A new graph-based clustering method with application to single-cell RNA-seq data from human pancreatic islets. NAR Genom. Bioinform. 2021; 3:lqaa087. 33575647 10.1093/nargab/lqaa087 PMC7803008

[14] Stuart T. , Butler A., Hoffman P., Hafemeister C., Papalexi E., Mauck W.M., Hao Y., Stoeckius M., Smibert P., Satija R. Comprehensive Integration of Single-Cell Data. Cell. 2019; 177:1888–1902. 31178118 10.1016/j.cell.2019.05.031 PMC6687398

[15] Wolf F.A. , Angerer P., Theis F.J. SCANPY: large-scale single-cell gene expression data analysis. Genome Biol. 2018; 19:15. 29409532 10.1186/s13059-017-1382-0 PMC5802054

[16] Chen G. , Ning B., Shi T. Single-cell RNA-seq technologies and related computational data analysis. Front Genet. 2019; 10:317. 31024627 10.3389/fgene.2019.00317 PMC6460256

[17] Haque A. , Engel J., Teichmann S.A., Lönnberg T. A practical guide to single-cell RNA-sequencing for biomedical research and clinical applications. Genome Med. 2017; 9:75. 28821273 10.1186/s13073-017-0467-4 PMC5561556

[18] Ziegenhain C. , Vieth B., Parekh S., Reinius B., Guillaumet-Adkins A., Smets M., Leonhardt H., Heyn H., Hellmann I., Enard W. Comparative Analysis of Single-Cell RNA Sequencing Methods. Mol. Cell. 2017; 65:631–643. 28212749 10.1016/j.molcel.2017.01.023

[19] Pullin J.M. , McCarthy D.J. A comparison of marker gene selection methods for single-cell RNA sequencing data. 2022; bioRxiv doi:10 May 2022, preprint: not peer reviewed 10.1101/2022.05.09.490241. PMC10895860 38409056

[20] Shim W.J. , Sinniah E., Xu J., Vitrinel B., Alexanian M., Andreoletti G., Shen S., Sun Y., Balderson B., Boix C. et al. Conserved Epigenetic Regulatory Logic Infers Genes Governing Cell Identity. Cell Syst. 2020; 11:625–639. 33278344 10.1016/j.cels.2020.11.001 PMC7781436

[21] Budiarto A. , Mahesworo B., Hidayat A.A., Nurlaila I., Pardamean B. Gaussian mixture model implementation for population stratification estimation from genomics data. Procedia Comput. Sci. 2021; 179:202–210.

[22] Prabakaran I. , Wu Z., Lee C., Tong B., Steeman S., Koo G., Zhang P.J., Guvakova M.A. Gaussian mixture models for probabilistic classification of breast cancer. Cancer Res. 2019; 79:3492–3502. 31113820 10.1158/0008-5472.CAN-19-0573

[23] Rafique O. , Mir A.H. Weighted dimensionality reduction and robust Gaussian mixture model based cancer patient subtyping from gene expression data. J. Biomed. Inform. 2020; 112:103620. 33188907 10.1016/j.jbi.2020.103620

[24] Ficklin S.P. , Dunwoodie L.J., Poehlman W.L., Watson C., Roche K.E., Feltus F.A. Discovering condition-specific gene co-expression patterns using Gaussian mixture models: a cancer case study. Sci. Rep. 2017; 7:8617. 28819158 10.1038/s41598-017-09094-4 PMC5561081

[25] Xu W. , Zhang X., Feng H. 2008 2nd International Conference on Bioinformatics and Biomedical Engineering. 2008; 470–473.

[26] Liu T.-C. , Kalugin P.N., Wilding J.L., Bodmer W.F. GMMchi: gene expression clustering using Gaussian mixture modeling. BMC Bioinf. 2022; 23:457. 10.1186/s12859-022-05006-0 PMC9632092 36324085

[27] Gao C. , Zhu Y., Shen X., Pan W. Estimation of multiple networks in Gaussian mixture models. Electron. J. Stat. 2016; 10:1133–1154. 28966702 10.1214/16-EJS1135 PMC5620020

[28] Langfelder P. , Horvath S. WGCNA: an R package for weighted correlation network analysis. BMC Bioinf. 2008; 9:559. 10.1186/1471-2105-9-559 PMC2631488 19114008

[29] Zhao J. , Jaffe A., Li H., Lindenbaum O., Sefik E., Jackson R., Cheng X., Flavell R.A., Kluger Y. Detection of differentially abundant cell subpopulations in scrna-seq data. Proc. Natl. Acad. Sci. U.S.A. 2021; 118:1. 10.1073/pnas.2100293118 PMC8179149 34001664

[30] Vandenbon A. , Diez D A clustering-independent method for finding differentially expressed genes in single-cell transcriptome data. Nat. Commun. 2020; 11:4318–4318. 32859930 10.1038/s41467-020-17900-3 PMC7455704

[31] Thalia E.C. , Michael S., Ann C.B. Gene regulatory network inference from single-cell data using multivariate information measures. Cell Systems. 2017; 5:251–267. 28957658 10.1016/j.cels.2017.08.014 PMC5624513

[32] Iacono G. , Massoni-Badosa R., Heyn H. Single-cell transcriptomics unveils gene regulatory network plasticity. Genome Biol. 2019; 20:110. 31159854 10.1186/s13059-019-1713-4 PMC6547541

[33] Jin S. , Zhang L., Nie Q. scAI: an unsupervised approach for the integrative analysis of parallel single-cell transcriptomic and epigenomic profiles. Genome Biol. 2020; 21:25. 32014031 10.1186/s13059-020-1932-8 PMC6996200

[34] Zhang M. , Liu S., Miao Z., Han F., Gottardo R., Sun W. IDEAS: individual level differential expression analysis for single-cell RNA-seq data. Genome Biol. 2022; 23:33. 35073995 10.1186/s13059-022-02605-1 PMC8784862

[35] Fatemeh Behjati A. , Kathrin K., Karl N., Nina G., Gilles G., Sarah F., Anupam S., Matthias B., Peter E., Jonas F. et al. Integrative analysis of single cell expression data reveals distinct regulatory states in bidirectional promoters. Epigenetics Chromatin. 2018; 11:66. 30414612 10.1186/s13072-018-0236-7 PMC6230222

[36] Kircher M. , Witten D.M., Jain P., O’Roak B.J., Cooper G.M., Shendure J. A general framework for estimating the relative pathogenicity of human genetic variants. Nat. Genet. 2014; 46:310–315. 24487276 10.1038/ng.2892 PMC3992975

[37] Mizikovsky D. , Naval Sanchez M., Nefzger C.M., Cuellar Partida G., Palpant N.J Organization of gene programs revealed by unsupervised analysis of diverse gene–trait associations. Nucleic Acids Res. 2022; 50:e87. 35716123 10.1093/nar/gkac413 PMC9410900

[38] Cuomo A.S.E. , Seaton D.D., McCarthy D.J., Martinez I., Bonder M.J., Garcia-Bernardo J., Amatya S., Madrigal P., Isaacson A., Buettner F. et al. Single-cell RNA-sequencing of differentiating iPS cells reveals dynamic genetic effects on gene expression. Nat. Commun. 2020; 11:810. 32041960 10.1038/s41467-020-14457-z PMC7010688

[39] Friedman C.E. , Nguyen Q., Lukowski S.W., Helfer A., Chiu H.S., Miklas J., Levy S., Suo S., Han J.-D.J., Osteil P. et al. Single-Cell Transcriptomic Analysis of Cardiac Differentiation from Human PSCs Reveals HOPX-Dependent Cardiomyocyte Maturation. Cell Stem Cell. 2018; 23:586–598. 30290179 10.1016/j.stem.2018.09.009 PMC6220122

[40] Kolde R. pheatmap: Pretty Heatmaps. R package version 1.0.12. 2019; https://CRAN.R-project.org/package=pheatmap.

[41] Pijuan-Sala B. , Griffiths J.A., Guibentif C., Hiscock T.W., Jawaid W., Calero-Nieto F.J., Mulas C., Ibarra-Soria X., Tyser R.C.V., Ho D.L.L. et al. A single-cell molecular map of mouse gastrulation and early organogenesis. Nature. 2019; 566:490–495. 30787436 10.1038/s41586-019-0933-9 PMC6522369

[42] McCarthy D.J. , Campbell K.R., Lun A.T.L., Wills Q.F. Scater: pre-processing, quality control, normalization and visualization of single-cell RNA-seq data in R. Bioinformatics. 2017; 33:1179–1186. 28088763 10.1093/bioinformatics/btw777 PMC5408845

[43] Lun A. , McCarthy D., Marioni J. A step-by-step workflow for low-level analysis of single-cell RNA-seq data with Bioconductor (version 2; peer review: 3 approved, 2 approved with reservations). F1000Research. 2016; 5:2122. 27909575 10.12688/f1000research.9501.1 PMC5112579

[44] Becht E. , McInnes L., Healy J., Dutertre C.-A., Kwok I.W.H., Ng L.G., Ginhoux F., Newell E.W. Dimensionality reduction for visualizing single-cell data using UMAP. Nat. Biotechnol. 2019; 37:38–44. 10.1038/nbt.4314 30531897

[45] Cao J. , Spielmann M., Qiu X., Huang X., Ibrahim D.M., Hill A.J., Zhang F., Mundlos S., Christiansen L., Steemers F.J. et al. The single-cell transcriptional landscape of mammalian organogenesis. Nature. 2019; 566:496–502. 30787437 10.1038/s41586-019-0969-x PMC6434952

[46] Guo L. , Lin L., Wang X., Gao M., Cao S., Mai Y., Wu F., Kuang J., Liu H., Yang J. et al. Resolving cell fate decisions during somatic cell reprogramming by single-cell RNA-Seq. Mol. Cell. 2019; 73:815–829. 30772174 10.1016/j.molcel.2019.01.042

[47] Wang X. , Allen W.E., Wright M.A., Sylwestrak E.L., Samusik N., Vesuna S., Evans K., Liu C., Ramakrishnan C., Liu J. et al. Three-dimensional intact-tissue sequencing of single-cell transcriptional states. Science. 2018; 361:eaat5691. 29930089 10.1126/science.aat5691 PMC6339868

[48] Jean-Baptiste K. , McFaline-Figueroa J.L., Alexandre C.M., Dorrity M.W., Saunders L., Bubb K.L., Trapnell C., Fields S., Queitsch C., Cuperus J.T. Dynamics of gene expression in single root cells of *Arabidopsis thaliana*. Plant Cell. 2019; 31:993–1011. 30923229 10.1105/tpc.18.00785 PMC8516002

[49] Saunders L.M. , Mishra A.K., Aman A.J., Lewis V.M., Toomey M.B., Packer J.S., Qiu X., McFaline-Figueroa J.L., Corbo J.C., Trapnell C. et al. Thyroid hormone regulates distinct paths to maturation in pigment cell lineages. Elife. 2019; 8:e45181. 31140974 10.7554/eLife.45181 PMC6588384

[50] Virtanen P. , Gommers R., Oliphant T.E., Haberland M., Reddy T., Cournapeau D., Burovski E., Peterson P., Weckesser W., Bright J. et al. SciPy 1.0: fundamental algorithms for scientific computing in Python. Nat. Methods. 2020; 17:261–272. 32015543 10.1038/s41592-019-0686-2 PMC7056644

[51] Scott D.W. Multivariate Density Estimation: Theory, Practice, and Visualization. 1992; NY Wiley.

[52] Pedregosa F. , Varoquaux G., Gramfort A., Michel V., Thirion B., Grisel O., Blondel M., Prettenhofer P., Weiss R., Dubourg V. et al. Scikit-learn: machine learning in Python. J. Mach. Learn. Res. 2011; 12:2825–2830.

[53] Chari T. , Pachter L. The specious art of single-cell genomics. 2022; bioRxiv doi:22 December 2022, preprint: not peer reviewed 10.1101/2021.08.25.457696. PMC10434946 37590228

[54] Hubert L. , Arabie P. Comparing partitions. J. Classification. 1985; 2:193–218.

[55] Scrucca L. , Fop M., Murphy T.B., Raftery A.E. mclust 5: clustering, classification and density estimation using Gaussian finite mixture models. R j. 2016; 8:289–317. 27818791 PMC5096736

[56] Kiselev V.Y. , Kirschner K., Schaub M.T., Andrews T., Yiu A., Chandra T., Natarajan K.N., Reik W., Barahona M., Green A.R. et al. SC3: consensus clustering of single-cell RNA-seq data. Nat. Methods. 2017; 14:483–486. 28346451 10.1038/nmeth.4236 PMC5410170

[57] van Dijk D. , Sharma R., Nainys J., Yim K., Kathail P., Carr A.J., Burdziak C., Moon K.R., Chaffer C.L., Pattabiraman D. et al. Recovering gene interactions from single-cell data using data diffusion. Cell. 2018; 174:716–729. 29961576 10.1016/j.cell.2018.05.061 PMC6771278

[58] Bernstein B.E. , Stamatoyannopoulos J.A., Costello J.F., Ren B., Milosavljevic A., Meissner A., Kellis M., Marra M.A., Beaudet A.L., Ecker J.R. et al. The NIH roadmap epigenomics mapping consortium. Nat. Biotechnol. 2010; 28:1045–1048. 20944595 10.1038/nbt1010-1045 PMC3607281

[59] Boix C.A. , James B.T., Park Y.P., Meuleman W., Kellis M. Regulatory genomic circuitry of human disease loci by integrative epigenomics. Nature. 2021; 590:300–307. 33536621 10.1038/s41586-020-03145-z PMC7875769

[60] Quinlan A.R. BEDTools: the Swiss-Army Tool for Genome Feature Analysis. Curr. Protoc. Bioinform. 2014; 47:11.12.11–11.12.34. 10.1002/0471250953.bi1112s47 PMC4213956 25199790

[61] Kent W.J. , Zweig A.S., Barber G., Hinrichs A.S., Karolchik D BigWig and BigBed: enabling browsing of large distributed datasets. Bioinformatics. 2010; 26:2204–2207. 20639541 10.1093/bioinformatics/btq351 PMC2922891

[62] Zhang Y. , Liu T., Meyer C.A., Eeckhoute J., Johnson D.S., Bernstein B.E., Nusbaum C., Myers R.M., Brown M., Li W. et al. Model-based analysis of ChIP-Seq (MACS). Genome Biol. 2008; 9:R137. 18798982 10.1186/gb-2008-9-9-r137 PMC2592715

[63] DeLaughter D.M. , Bick A.G., Wakimoto H., McKean D., Gorham J.M., Kathiriya I.S., Hinson J.T., Homsy J., Gray J., Pu W. et al. Single-cell resolution of temporal gene expression during heart development. Dev. Cell. 2016; 39:480–490. 27840107 10.1016/j.devcel.2016.10.001 PMC5198784

[64] Szklarczyk D. , Gable A.L., Lyon D., Junge A., Wyder S., Huerta-Cepas J., Simonovic M., Doncheva N.T., Morris J.H., Bork P. et al. STRING v11: protein–protein association networks with increased coverage, supporting functional discovery in genome-wide experimental datasets. Nucleic Acids Res. 2019; 47:D607–D613. 30476243 10.1093/nar/gky1131 PMC6323986

[65] Blagus R. , Lusa L. SMOTE for high-dimensional class-imbalanced data. BMC Bioinf. 2013; 14:106. 10.1186/1471-2105-14-106 PMC3648438 23522326

[66] Kuppe C. , Ramirez Flores R.O., Li Z., Hayat S., Levinson R.T., Liao X., Hannani M.T., Tanevski J., Wünnemann F., Nagai J.S. et al. Spatial multi-omic map of human myocardial infarction. Nature. 2022; 608:766–777. 35948637 10.1038/s41586-022-05060-x PMC9364862

[67] Li G. , Xu A., Sim S., Priest J.R., Tian X., Khan T., Quertermous T., Zhou B., Tsao P.S., Quake S.R et al. Transcriptomic profiling maps anatomically patterned subpopulations among single embryonic cardiac cells. Dev. Cell. 2016; 39:491–507. 27840109 10.1016/j.devcel.2016.10.014 PMC5130110

[68] Sim C.B. , Phipson B., Ziemann M., Rafehi H., Mills R.J., Watt K.I., Abu-Bonsrah K.D., Kalathur R.K.R., Voges H.K., Dinh D.T. et al. Sex-specific control of human heart maturation by the progesterone receptor. Circulation. 2021; 143:1614–1628. 33682422 10.1161/CIRCULATIONAHA.120.051921 PMC8055196

[69] Nicin L. , Abplanalp W.T., Schänzer A., Sprengel A., John D., Mellentin H., Tombor L., Keuper M., Ullrich E., Klingel K. et al. Single nuclei sequencing reveals novel insights into the regulation of cellular signatures in children with dilated cardiomyopathy. Circulation. 2021; 143:1704–1719. 33618539 10.1161/CIRCULATIONAHA.120.051391

[70] Tyser R.C.V. , Ibarra-Soria X., McDole K., Arcot Jayaram S., Godwin J., van den Brand T.A.H., Miranda A.M.A., Scialdone A., Keller P.J., Marioni J.C. et al. Characterization of a common progenitor pool of the epicardium and myocardium. Science. 2021; 371:eabb2986. 33414188 10.1126/science.abb2986 PMC7615359

[71] Shen S. , Werner T., Sun Y., Shim W.J., Lukowski S., Andersen S., Chiu H.S., Xia D., Chen X., Pham D. et al. An integrated cell barcoding and computational analysis pipeline for scalable analysis of differentiation at single-cell resolution. 2022; bioRxiv doi:27 October 2022, preprint: not peer reviewed 10.1101/2022.10.12.511862.

[72] Asp M. , Giacomello S., Larsson L., Wu C., Fürth D., Qian X., Wärdell E., Custodio J., Reimegård J., Salmén F. et al. A spatiotemporal organ-wide gene expression and cell atlas of the developing human heart. Cell. 2019; 179:1647–1660. 31835037 10.1016/j.cell.2019.11.025

[73] Lescroart F. , Wang X., Lin X., Swedlund B., Gargouri S., Sànchez-Dànes A., Moignard V., Dubois C., Paulissen C., Kinston S. et al. Defining the earliest step of cardiovascular lineage segregation by single-cell RNA-seq. Science. 2018; 359:1177–1181. 29371425 10.1126/science.aao4174 PMC6556615

[74] de Soysa T.Y. , Ranade S.S., Okawa S., Ravichandran S., Huang Y., Salunga H.T., Schricker A., del Sol A., Gifford C.A., Srivastava D Single-cell analysis of cardiogenesis reveals basis for organ-level developmental defects. Nature. 2019; 572:120–124. 31341279 10.1038/s41586-019-1414-x PMC6719697

[75] Han X. , Zhou Z., Fei L., Sun H., Wang R., Chen Y., Chen H., Wang J., Tang H., Ge W. et al. Construction of a human cell landscape at single-cell level. Nature. 2020; 581:303–309. 32214235 10.1038/s41586-020-2157-4

[76] Stassen S.V. , Yip G.G.K., Wong K.K.Y., Ho J.W.K., Tsia K.K. Generalized and scalable trajectory inference in single-cell omics data with VIA. Nat. Commun. 2021; 12:5528. 34545085 10.1038/s41467-021-25773-3 PMC8452770

[77] Boyer L.A. , Plath K., Zeitlinger J., Brambrink T., Medeiros L.A., Lee T.I., Levine S.S., Wernig M., Tajonar A., Ray M.K. et al. Polycomb complexes repress developmental regulators in murine embryonic stem cells. Nature. 2006; 441:349–353. 16625203 10.1038/nature04733

[78] Mikkelsen T.S. , Hanna J., Zhang X., Ku M., Wernig M., Schorderet P., Bernstein B.E., Jaenisch R., Lander E.S., Meissner A. Dissecting direct reprogramming through integrative genomic analysis. Nature. 2008; 454:49–55. 18509334 10.1038/nature07056 PMC2754827

[79] Pérez-Lluch S. , Blanco E., Tilgner H., Curado J., Ruiz-Romero M., Corominas M., Guigó R. Absence of canonical marks of active chromatin in developmentally regulated genes. Nat. Genet. 2015; 47:1158–1167. 26280901 10.1038/ng.3381 PMC4625605

[80] Kundaje A. , Meuleman W., Ernst J., Bilenky M., Yen A., Heravi-Moussavi A., Kheradpour P., Zhang Z., Wang J., Ziller M.J. et al. Integrative analysis of 111 reference human epigenomes. Nature. 2015; 518:317–330. 25693563 10.1038/nature14248 PMC4530010

[81] Ernst J. , Kellis M. Chromatin-state discovery and genome annotation with ChromHMM. Nat. Protoc. 2017; 12:2478–2492. 29120462 10.1038/nprot.2017.124 PMC5945550

[82] Paige S.L. , Thomas S., Stoick-Cooper C.L., Wang H., Maves L., Sandstrom R., Pabon L., Reinecke H., Pratt G., Keller G. et al. A temporal chromatin signature in human embryonic stem cells identifies regulators of cardiac development. Cell. 2012; 151:221–232. 22981225 10.1016/j.cell.2012.08.027 PMC3462257

[83] Griffiths J L.A. R Package Version 1.10.0. 2022;

