# Supplementary material for: Inferring cell diversity in single cell data using consortium-scale epigenetic data as a biological anchor for cell identity
Source: Nucleic Acids Res. 2023 May 1;51(11):e62. doi: 10.1093/nar/gkad307 (PMC10287941; doi:10.1093/nar/gkad307)
Supplement: gkad307_Supplemental_Files [file gkad307_supplemental_files.zip › Supplemental Information.pdf]

Supplemental Figures

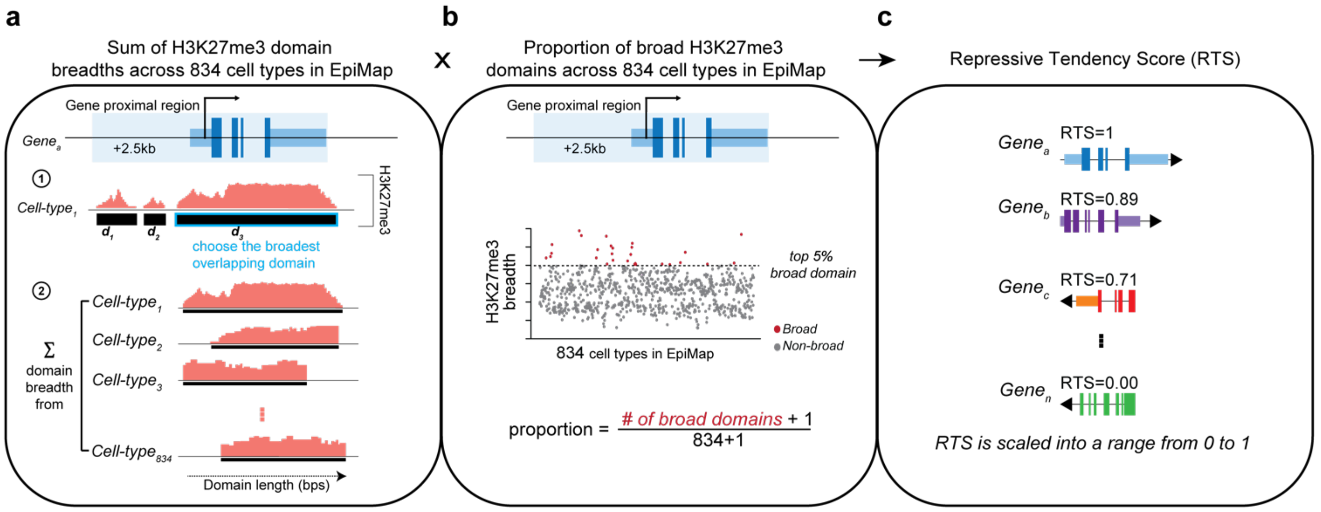

Figure S1. Schematic of domain assignment to genes and proportion of domain across 834 cell types in EpiMap data.

a-c) We begin by defining the H3K27me3 broad domain that overlaps with each gene in every cell type. We then calculate the total length of the broad domain in base pairs and scale the sum of breadth scores into a range of 0-1 **(a)**. Next, we multiply the total length of the broad domain by the proportion of cell types where the gene's H3K27me3 breadth is among the top 5% of broad domains **(b)**. This new score is also rescaled into a range of 0-1, representing the gene's repressive tendency score (RTS) **(c)**.

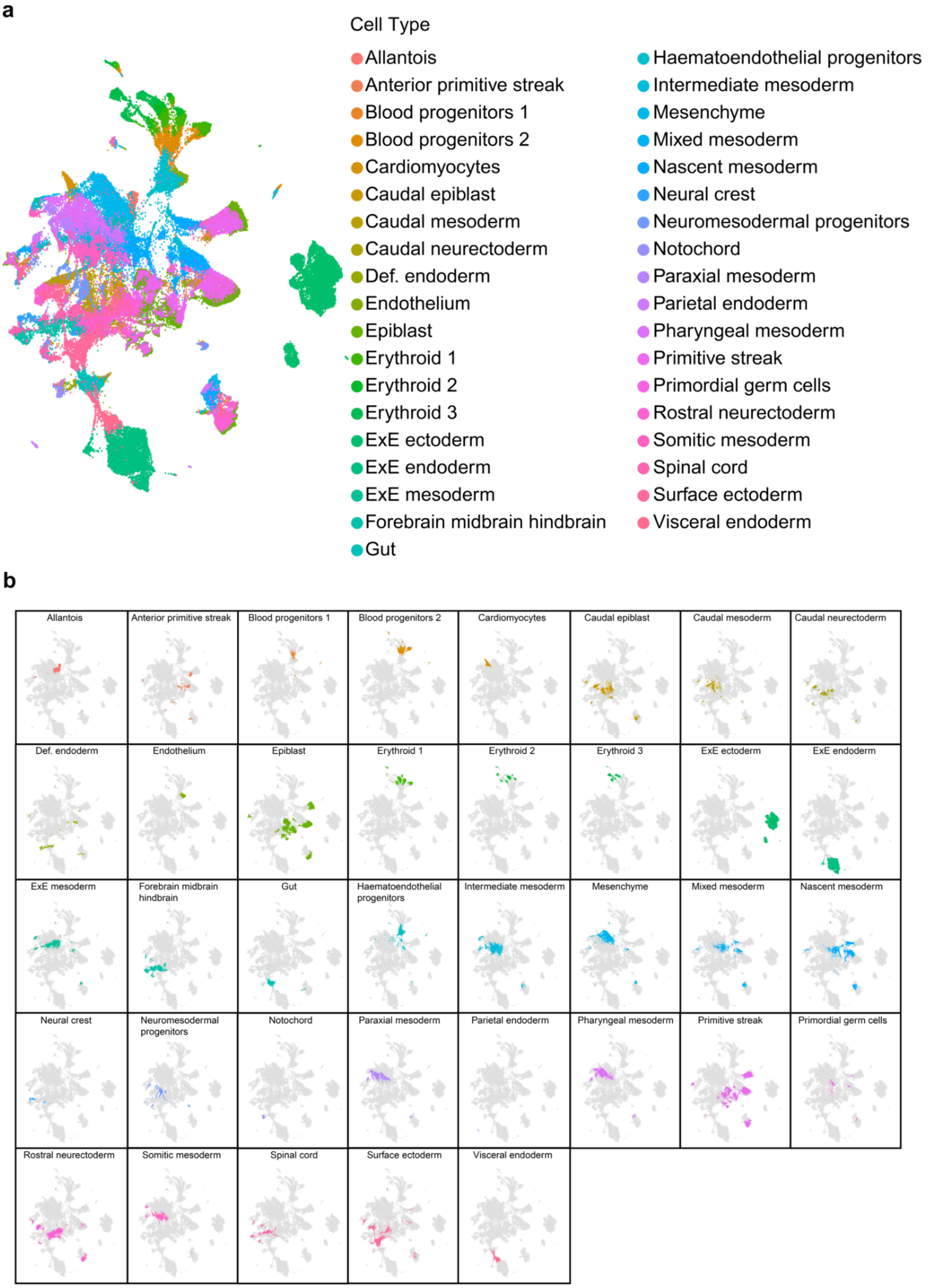

Figure S2. Overview of original annotation for the mouse gastrulation atlas data.

a-b) Original annotations are shown in single UMAP and separate in multiple UMAP plots.

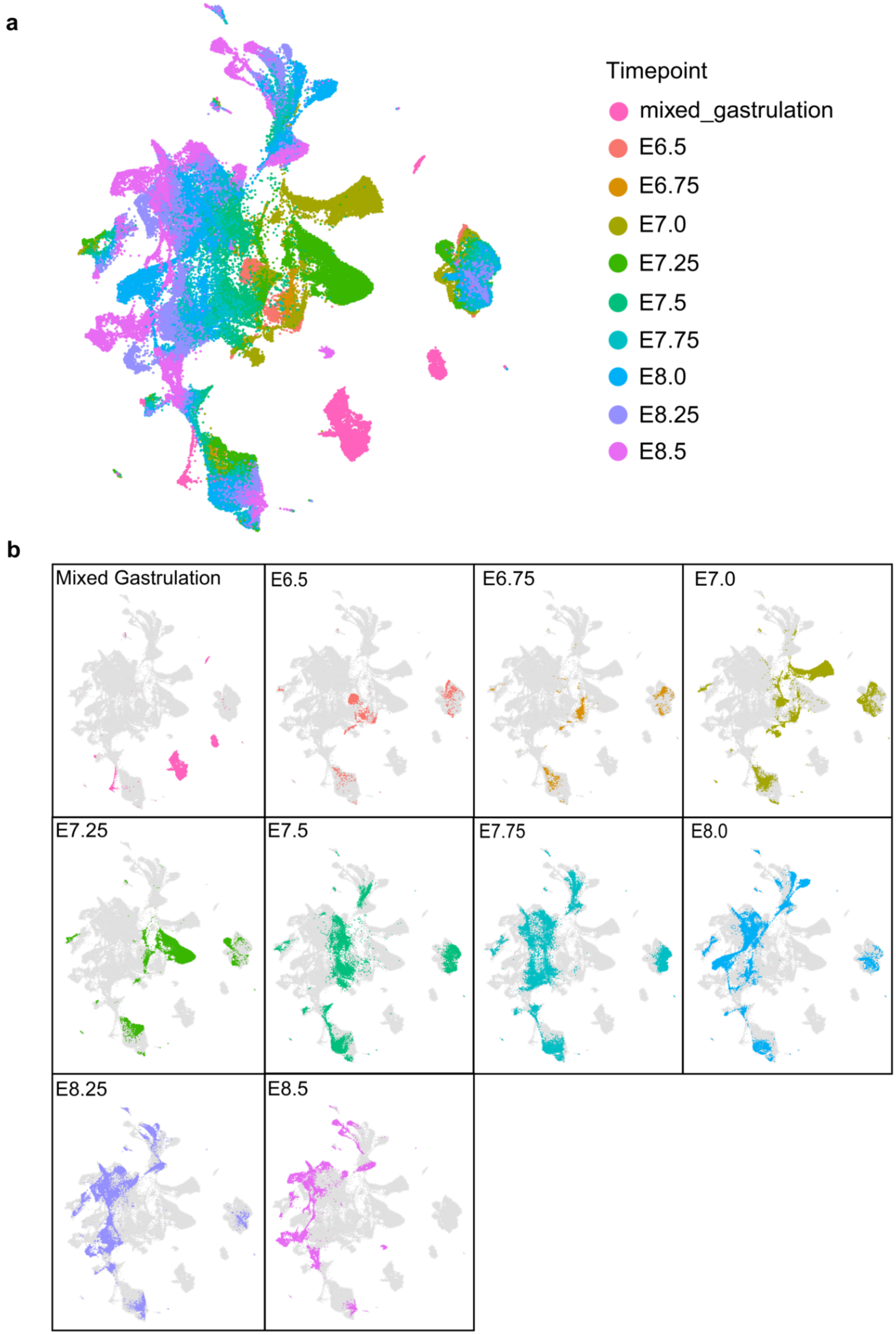

Figure S3. Overview of time point for the mouse gastrulation atlas data.

a-b) Time points are shown in single UMAP and separate in multiple UMAP plots.

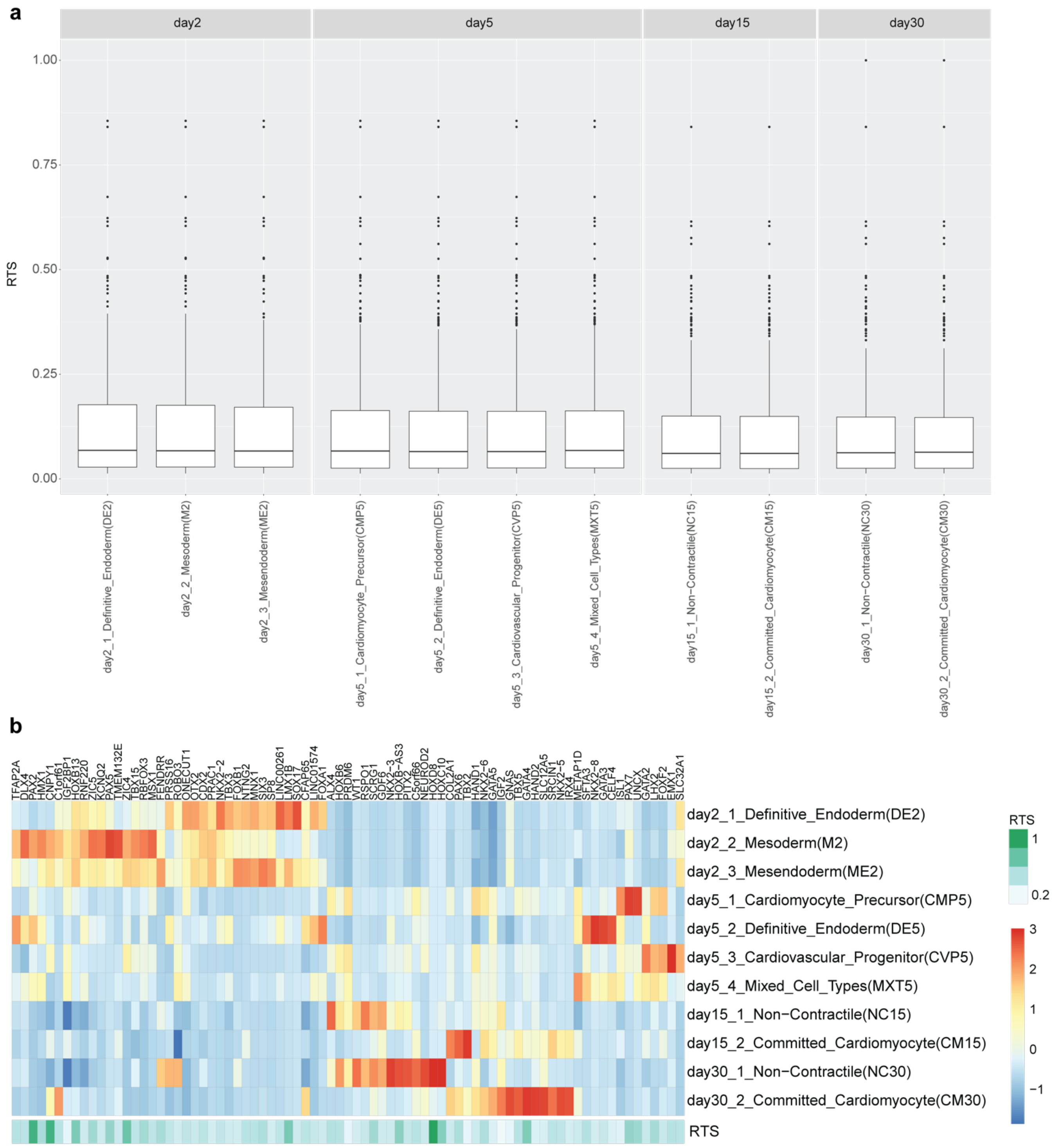

Figure S4. Evaluation of RTS values for lineage development and cell type identification.

a-b) We used a single-cell time-course of cardiac differentiation (1) to evaluate the association between gene RTS values at each stage of differentiation **(a)** and analysis of anchor genes defining TRIAGE-Cluster cell peaks across each time point **(b)**.

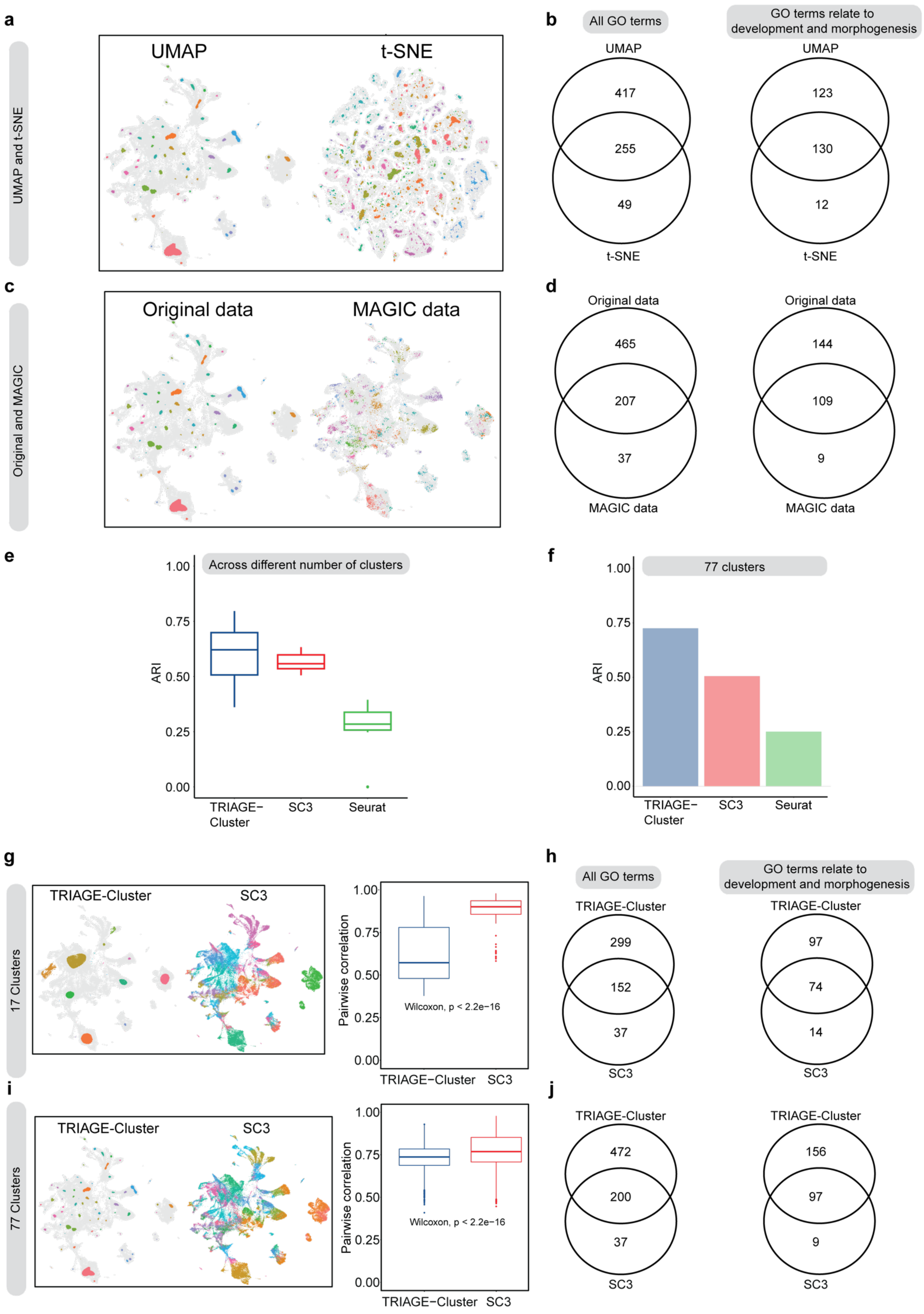

Figure S5. Performance testing of TRIAGE-Cluster.

a-b) GO enrichment analysis indicates that the peaks generated using UMAP are better at capturing differences in cell diversity in scRNA-seq datasets compared to t-SNE.

c-d) GO enrichment analysis showing that the original mouse gastrulation data captures differences in cell diversity compared to the MAGIC-imputed version of the data.

e-f) Performance test comparing TRIAGE-Cluster with Seurat and SC3 using an Adjusted Rand Index (ARI) across different cluster numbers **(e)** and at a specific cluster resolution for each method (77 clusters, **f**).

g-j) TRIAGE-Cluster captures greater cell diversity than SC3 as measured by Spearman rank correlation **(g, i)** and GO enrichment analysis **(h, j)**.

a

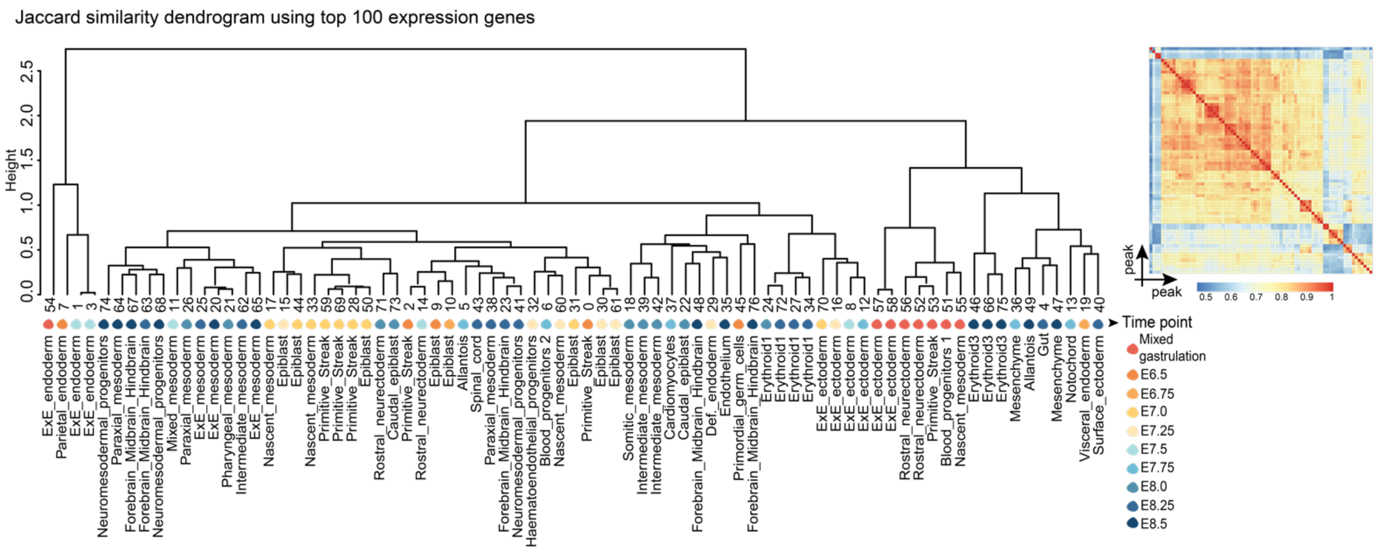

b

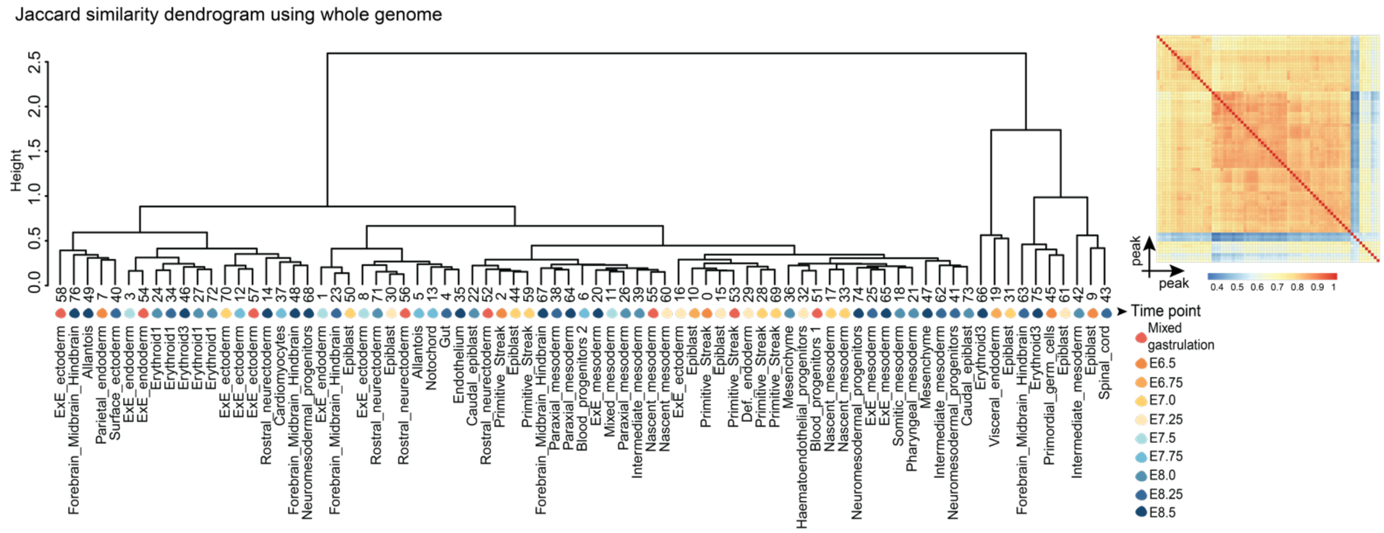

Figure S6. Comparing cell type identification using different gene lists.

a-b) Jaccard similarity dendrogram using top 100 genes ranked by original expression **(a)** and all genes expressed in each peak **(b)** at bandwidth 0.3 showing peak relationship (first row), mapped timepoint (second row), anchor gene (third row), and mapped original annotation (forth row).

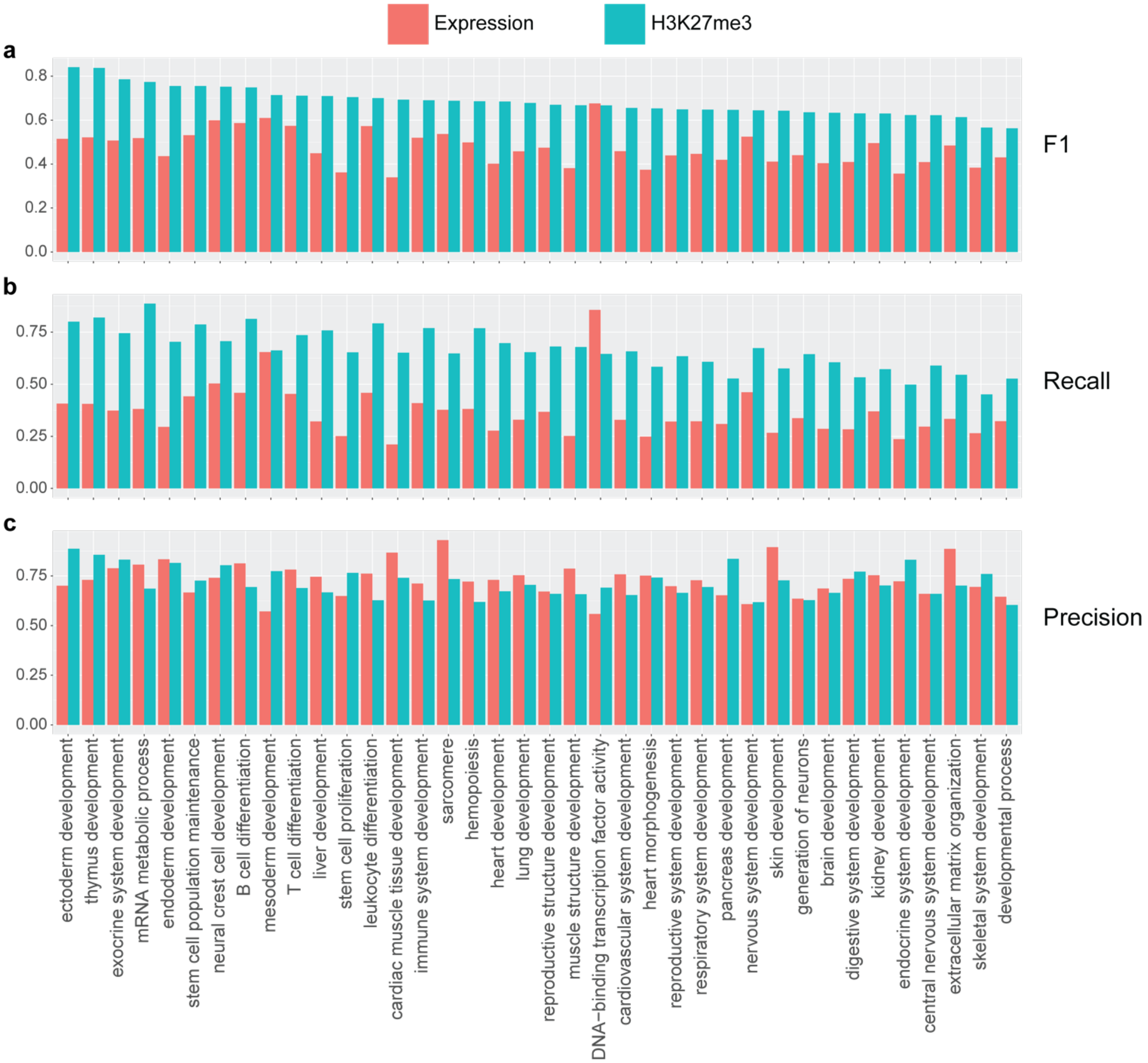

Figure S7. Performance analysis comparing the predictive power of gene expression and H3K27me3 breadth as features to predict genes with different biological processes, indicated by **(a)** F-score, **(b)** recall and **(c)** precision. H3K27me3 breadth is superior to the gene expression in recovering genes with various developmental gene ontology terms.

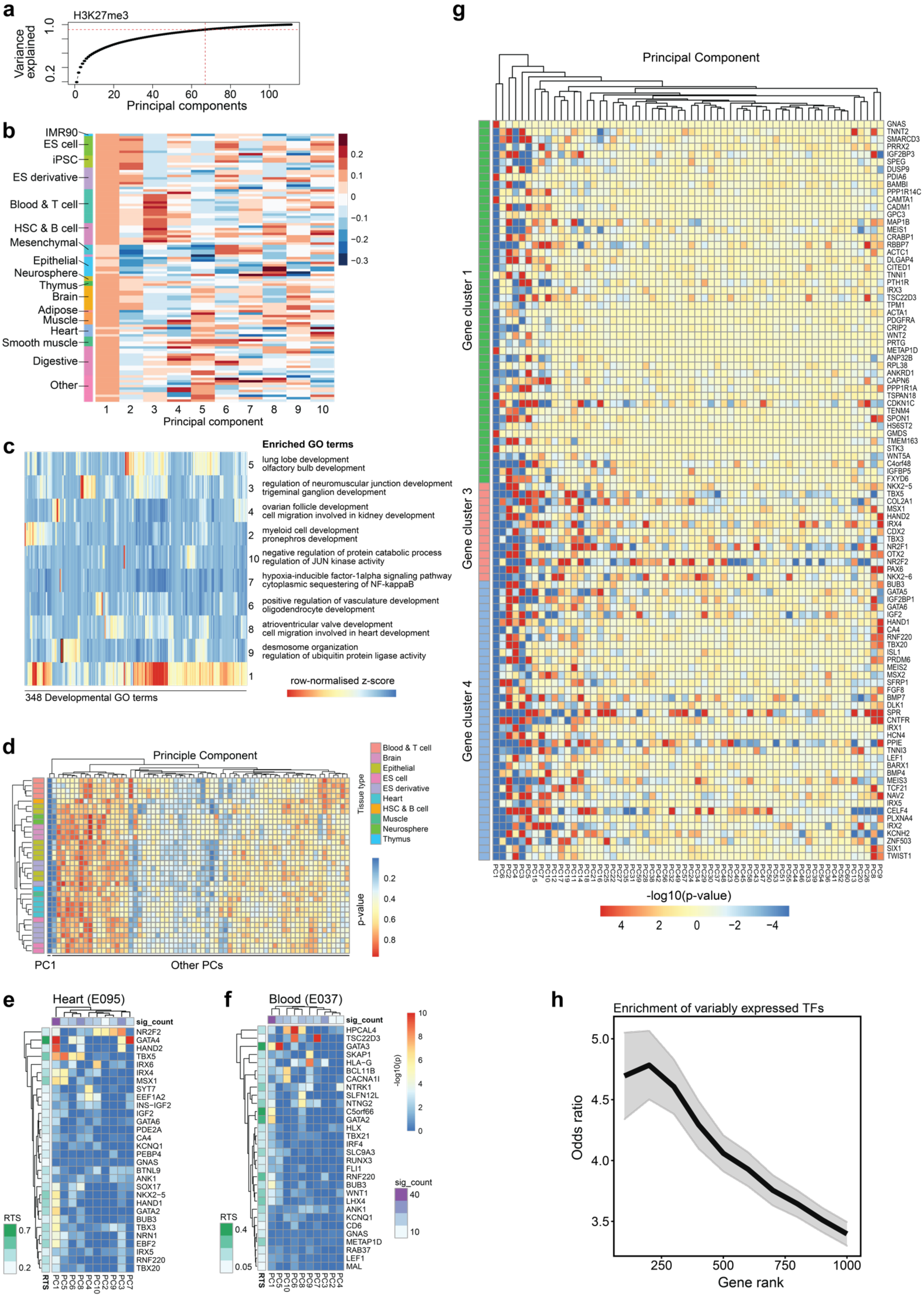
a
H3K27me3
Variance explained
Principal components
b
IMR90
ES cell
iPSC
ES derivative
Blood & T cell
HSC & B cell
Mesenchymal
Epithelial
Neurosphere
Thymus
Brain
Adipose
Muscle
Heart
Smooth muscle
Digestive
Other
Principal component
c
Enriched GO terms
lung lobe development
olfactory bulb development
regulation of neuromuscular junction development
trigeminal ganglion development
ovarian follicle development
cell migration involved in kidney development
myeloid cell development
pronephros development
negative regulation of protein catabolic process
regulation of JUN kinase activity
hypoxia-inducible factor-1alpha signaling pathway
cytoplasmic sequestering of NF-kappaB
positive regulation of vasculature development
oligodendrocyte development
atrioventricular valve development
cell migration involved in heart development
desmosome organization
regulation of ubiquitin protein ligase activity
row-normalised z-score
348 Developmental GO terms
d
Principle Component
Tissue type
Blood & T cell
Brain
Epithelial
ES cell
ES derivative
Heart
HSC & B cell
Muscle
Neurosphere
Thymus
p-value
PC1
Other PCs
e
Heart (E095)
sig_count
NR2F2
GATA4
HAND2
TBX5
IRX6
IRX4
MSX1
SYT7
EEF1A2
INS−IGF2
IGF2
GATA6
PDE2A
CA4
KCNQ1
PEBP4
GNAS
BTNL9
ANK1
SOX17
NKX2−5
HAND1
GATA2
BUB3
TBX3
NRN1
EBF2
IRX5
RNF220
TBX20
RTS
f
Blood (E037)
sig_count
HPCAL4
TSC22D3
GATA3
SKAP1
HLA−G
BCL11B
CACNA1I
NTRK1
SLFN12L
NTNG2
C5orf66
GATA2
HLX
TBX21
IRF4
SLC9A3
RUNX3
FLI1
RNF220
BUB3
WNT1
LHX4
ANK1
KCNQ1
CD6
GNAS
METAP1D
RAB37
LEF1
MAL
RTS
-log10(p)
g
Principal Component
Gene cluster 1
Gene cluster 3
Gene cluster 4
GNAS
TNNT2
SMARCD3
PRRX2
IGF2BP3
SPEG
DUSP9
PDIA6
BAMBI
PPP1R14C
CAMTA1
CADM1
GPC3
MAP1B
MEIS1
CRABP1
RBBP7
ACTC1
DLGAP4
CITED1
TNNI1
PTH1R
IRX3
TSC22D3
TPM1
ACTA1
PDGFRA
CRIP2
WNT2
PRTG
METAP1D
ANP32B
RPL38
ANKRD1
CAPN6
PPP1R1A
TSPAN18
CDKN1C
TENM4
SPON1
HS6ST2
GMDS
TMEM163
STK3
WNT5A
C4orf48
IGFBP5
FXYD6
NKX2−5
TBX5
COL2A1
MSX1
HAND2
IRX4
CDX2
TBX3
NR2F1
OTX2
NR2F2
PAX6
NKX2−6
BUB3
GATA5
IGF2BP1
GATA6
IGF2
HAND1
CA4
RNF220
TBX20
ISL1
PRDM6
MEIS2
MSX2
SFRP1
FGF8
BMP7
DLK1
SPR
CNTFR
IRX1
HCN4
PPIE
TNNI3
LEF1
BARX1
BMP4
MEIS3
TCF21
NAV2
IRX5
CELF4
PLXNA4
IRX2
KCNH2
ZNF503
SIX1
TWIST1
-log10(p-value)
h
Enrichment of variably expressed TFs
Odds ratio
Gene rank

Figure S8. H3K27me3 patterns mark genes regulating specific biological process.

a) Principal component analysis (PCA) showing approximately 96.5% H3K27me3 variance can be explained by top 67 PCs in Roadmap data (red dashed line).

b) The heatmap shows enrichment of the genes with top 100 discordance score from each sample in Roadmap in each PC.

c) The heatmap shows the Gene Ontology (GO) functional enrichment of 348 terms in each PC.

d) Enrichment of H3K27me3 patterns among top 100 RTS priority genes in Roadmap samples. The heatmap shows an enrichment p-value of a given pattern (or PC) using Fisher's exact test (one-tailed).

e-f) Enrichment of H3K27me3 patterns among top 30 RTS priority genes in left ventricle heart sample (E095) and blood sample (E037). Significant count is the number of genes among top 100 RTS priority genes showing significant enrichment of a given pattern. The heatmap shows the enrichment p-value (as -log10(p)) of the gene.

g) The heatmap shows an enrichment of H3K27me3 patterns among top 100 RTS priority genes using Fisher's exact test (one-tailed) in the three gene clusters detected in cardiomyocytes from the mouse gastrulation atlas data.

h) Enrichment of variably expressed TFs in top genes ranked by discordance score. Average odds ratio of variably expressed (defined by expression coefficient of variation>1) TFs across 46 different cell and tissue types from NIH Roadmap data were shown, with +/- standard error of the mean.

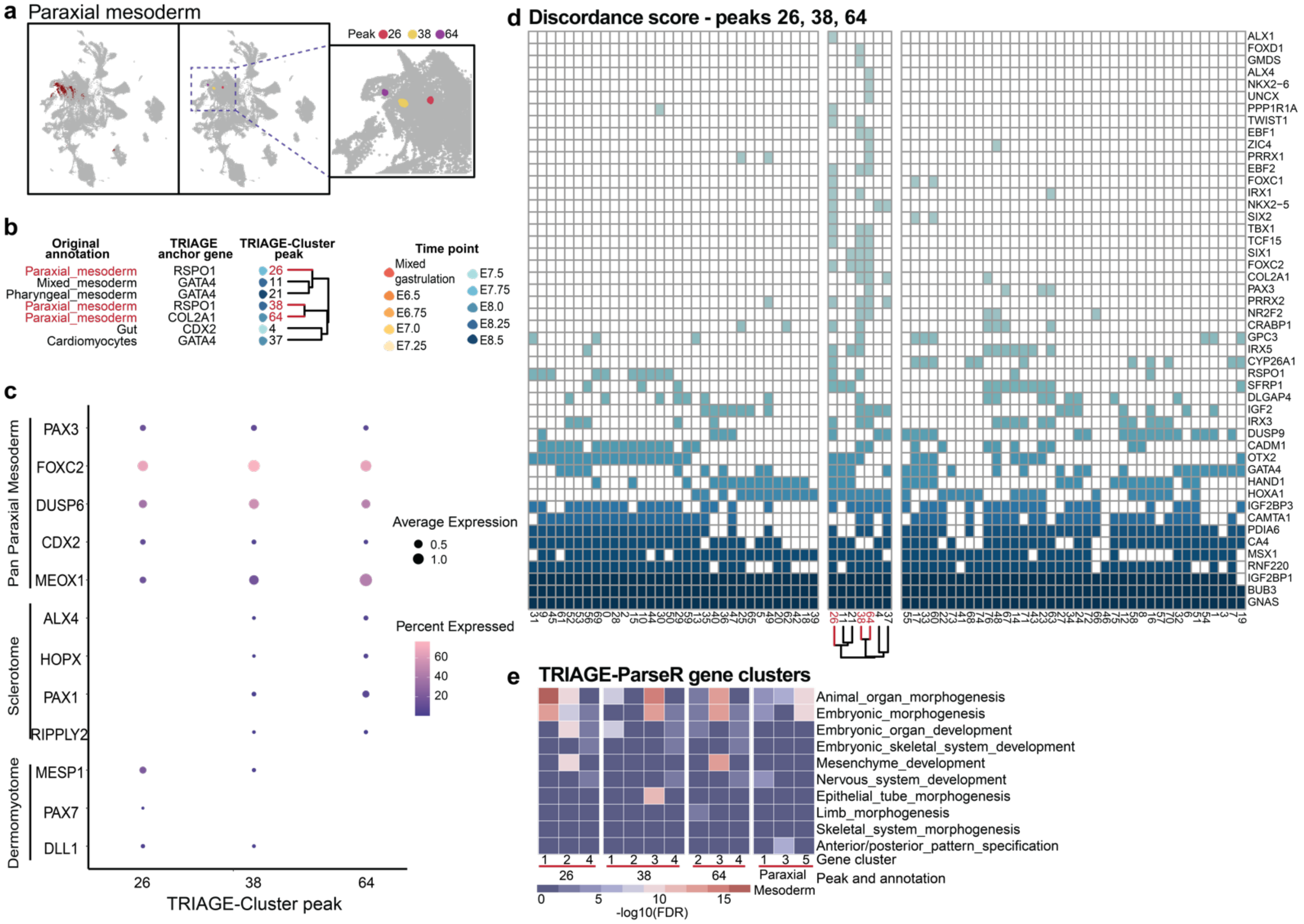

Figure S9. TRIAGE-Cluster and TRIAGE-ParseR analysis enable identification of cell subtype diversity in single cell data.

a) UMAP of original annotation paraxial mesoderm (left) and mapped TRIAGE-Cluster peaks (right and inset).

b) Jaccard similarity dendrogram (below) with bandwidth 0.3 showing three distinct "paraxial mesoderm" TRIAGE-Cluster peaks with their respective mapped timepoint and anchor genes (extracted from **Figure 2h**).

c) Dot plot showing marker genes associated with bone development lineage subtypes in each paraxial mesoderm peak.

d) Genes ranked by discordance score for each paraxial mesoderm peak (red) and measured across all other peaks. Output focuses on genes enriched in paraxial mesoderm peaks (row) for each TRIAGE-Cluster peak (column) to facilitate cell subtype classification.

e) Heatmap showing skeletal cell subtype gene ontology enrichment (-log10 (FDR)) from PCA-GMM analysis across gene clusters in the three paraxial mesoderm peaks (the first three blocks) and original paraxial mesoderm cells (the last block).

Supplemental Tables

Table S1. Gene RTS scores.

Table S2. EpiMap data.

Table S3. Composition of peaks in each original annotation in mouse embryonic data.

Table S4. Composition of peaks in each original annotation in human myocardial infarction data.

Table S5. Single-cell datasets of *in vivo* development and *in vitro* stem cell differentiation.

Table S6. Gene Ontology for 17 peaks from TRIAGE-Cluster.

Table S7. Gene Ontology for 77 peaks from TRIAGE-Cluster.

Table S8. Gene Ontology for 17 peaks from Seurat clustering.

Table S9. Gene Ontology for 77 peaks from Seurat clustering.

Table S10. Software details.

Table S5. Single-cell datasets of *in vivo* development and *in vitro* stem cell differentiation.

Species	Legend in Figure 4b	Timepoints	Lineages	Cell number	Data format	Platform	Source	Refs
in vivo Mouse	Cao *et al.*	E9.5<br>E10.5<br>E11.5<br>E12.5<br>E13.5	Connective Tissue Progenitors	2 million cells	Raw count	sci-RNA-seq3	GSE119945	(2)
			Chondrocytes and Osteoblasts					
			Intermediate Mesoderm					
			Jaw and Tooth Progenitors					
			Excitatory Neurons					
			Epithelial Cells					
			Radial Glia					
			Early Mesenchyme					
			Neural Progenitor Cells					
			Postmitotic Premature Neurons					
			Oligodendrocyte Progenitors					
			Isthmic Organizer Cells					
			Myocytes					
			Neural Tube					
			Stromal Cells					
			Osteoblasts					
			Inhibitory Neuron Progenitors					
			Premature Oligodendrocyte					
			Endothelial Cells					
			Chondrocyte Progenitors					
			Definitive Erythroid Lineage					

			Schwann Cell Precursor					
			Sensory Neurons					
			Limb Mesenchyme					
			Primitive Erythorid Lineage					
			Inhibitory interneurons					
			Granule Neurons					
			Hepatocytes					
			Notochord Cells					
			White Blood Cells					
			Ependymal Cell					
			Cholinergic Neurons					
			Cardiac Muscle Lineage					
			Megakaryocytes					
			Melanocytes					
			Lens					
			Neutrophils					
			Inhibitory Neurons					
in vivo Mouse	Lescroart *et al.*	E6.5<br>E6.75<br>E7.25<br>E7.5	Epiblast Cells<br>Endothelial Cells<br>Anterior Second Heart Field or Pharyngeal Mesoderm<br>Cardiomyocytes or Mesenchymal<br>Notochord or Definitive Endoderm<br>Posterior Second Heart Field or Somatic Mesoderm<br>Others	892 cells	Raw Count	SMART-seq2	GSE100471, http://singlecell.stemcells.cam.ac.uk/mesp1	(3)

in vivo Mouse	Li *et al.*	E8.5<br>E9.5<br>E10.5	Cardiomyocytes<br>Endothelial Cells<br>Mesenchymal Cells<br>Epicardial Cells	3,241 cells	Raw Count	Fluidigm C1	GSE76118	(4)
in vivo Mouse	DeLaughter *et al.*	E9.5<br>E11.5<br>E14.5<br>E18.5<br>P0<br>P3<br>P7<br>P21	Cardiomyocytes<br>Endothelial Cells<br>Fibroblast Cells	1,498 cells	Normalised	Fluidigm C1	Request from author	(5)
in vivo Mouse	Pijuan-Sala *et al.*(Figure 4a)<br>(mouse gastrulation atlas data used in the whole pipeline)	E6.5<br>E6.75<br>E7.0<br>E7.25<br>E7.5<br>E7.75<br>E8.0<br>E8.25<br>mixed gastrulation	Epiblast	113,126 cells	Raw Count	10x Genomics	Bioconductor Package MouseGastrulationData	(6)
			Primitive_Streak					
			ExE_ectoderm					
			ExE_endoderm					
			ExE_mesoderm					
			Visceral_endoderm					
			Nascent_mesoderm					
			Rostral_neurectoderm					
			Mixed_mesoderm					
			Surface_ectoderm					
			Intermediate_mesoderm					
			Pharyngeal_mesoderm					
			Caudal_epiblast					
			Primordial_germ_cells					
			Mesenchyme					
			Haematoendothelial_progenitors					
			Blood_progenitors 1					

			Blood_progenitors 2					
			Gut					
			Paraxial_mesoderm					
			Caudal_neurectoderm					
			Notochord					
			Somitic_mesoderm					
			Caudal_Mesoderm					
			Erythroid1					
			Erythroid2					
			Erythroid3					
			Def._endoderm					
			Allantois					
			Parietal_endoderm					
			Anterior_Primitive Streak					
			Endothelium					
			Forebrain/Midbrain/Hindbrain					
			Spinal_cord					
			Cardiomyocytes					
			Neuromesodermal_progenitors					
			Neural_crest					
in vivo Mouse	de Soysa *et al.*	E7.75 E8.25 E9.25	Endoderm_E7.75_1	25,122 cells	Raw Count	10x Genomics	UCSC Cell Browser: https://mouse-cardiac.cells.ucsc.edu	(7)
			Endoderm_E7.75_2					
			Endoderm_E7.75_3					
			Endoderm_E7.75_4					
			Endoderm_E7.75_5					

			Endocardial/Endothelial					
			Hematoendothelial					
			Endothelial_Mesenchymal_Transition					
			Anterior_Heart_Field					
			Posterior_Second_Heart_Field					
			Branchiomeric_Muscle_Progenitors					
			embryonic_myocardium_progenitors					
			Ventricle					
			Sinus_Venosus					
			Atrial					
			Outflow_Tract					
			Atrioventricular_Canal					
			Left_Ventricle					
			EarlyRV					
			Right_Ventricle					
in vivo Mouse	Tyser *et al.*	E7.75<br>E8.25	Mesodermal<br>Endothelial<br>Blood<br>Definitive endoderm<br>Yolk sac<br>Surface/amnion ectoderm<br>Neurectoderm	3,104 cells	Raw Count	SMART-seq2	https://content.cruk.cam.ac.uk/jmlab/mouseEmbryonicHeartAtlas/	(8)
in vivo Human	Asp *et al.*	PCW 4.5-5 (4 replicates)<br>PCW 6.5 (9 replicates)	Erythrocytes	Spatial transcriptomics: PCW 4.5-5 (55 spots)	Raw Count	ST: Spatial Transcriptomics	https://www.spatialresearch.org	(9)
			Epicardium-derived Cells					
			Capillary Endothelium					

		PCW 9 (6 replicates)	Smooth muscle cells / Fibroblast-like	PCW 6.5 (104 spots)<br>PCW 9 (238 spots)<br>scRNA-seq: 3,777 cells		SC: 10x Genomics		
			Endothelium / Pericytes / Adventia					
			Myoz2-enriched Cardiomyocytes					
			Immune cells					
			Epicardial Cells					
			Ventricular Cardiomyocytes					
			Atrial Cardiomyocytes					
			Cardiac neural crest / Schwann Progenitor Cells					
			Fibroblast-like (related to cardiac skeleton connective tissue)					
			Fibroblast-like (related to smaller vascular development)					
			Fibroblast-like (related to larger vascular development)					
in vivo Human	Nicin *et al.*	0.5y<br>0.75y<br>5y<br>6y<br>12y<br>13y	Endothelial_cell1	18,211 cells	Raw Count	10x Genomics	Request from author	(10)
			Endothelial_cell2					
			Endothelial_cell3					
			Cardiomyocyte1					
			Cardiomyocyte2					
			Cardiomyocyte3					
			Fibroblast					
			Leukocyte					
			Pericyte					
			Smooth_muscle_cell					
			Endothelial_fibroblast_like					

			Neuronal_like_cell					
in vivo<br>Human	Sim *et al.*	9 human donors from<br>fetal satges to adulthood	Cardiomyocytes<br>Fibroblasts<br>Endothelial_cells<br>Epicardial_cells<br>Immune_cells<br>Smooth_muscle_cells<br>Neural_cells<br>Erythroid_cells	37,089 cells	Raw Count	10x Genomics	Request from author	(11)
in vivo<br>Human	Han *et al.*	60 human tissue from both fetal and adult	Fetal brain<br>Fetal calvaria<br>Fetal eyes<br>Fetal female gonad<br>Fetal heart<br>Fetal intestine<br>Fetal kidney<br>Fetal liver<br>Fetal lung<br>Fetal male gonad<br>Fetal muscle<br>Fetal pancreas<br>Fetal rib<br>Fetal skin<br>Fetal spinal cord<br>Fetal stomach<br>Fetal thymus<br>Human ES cells<br>Neonatal adrenal gland<br>Placenta<br>Adult omentum<br>Adult pancreas<br>Adult peripheral blood<br>Adult pleura<br>Adult prostate<br>Adult rectum<br>Adult sigmoid colon<br>Adult spleen<br>Adult stomach<br>Adult adipose<br>Adult adrenal gland<br>Adult artery	702,698 cells	Raw Count	Microwell-seq	GSE134355	(12)

			Adult ascending colon<br>Adult bladder<br>Adult bone marrow<br>Adult cerebellum<br>Adult cervix<br>Adult duodenum<br>Adult epityphlon<br>Adult oesophagus<br>Adult fallopian tube<br>Adult gall bladder<br>Adult heart<br>Adult ileum<br>Adult jejunum<br>Adult kidney<br>Adult liver<br>Adult lung<br>Adult muscle<br>Adult temporal lobe<br>Adult thyroid gland<br>Adult trachea<br>Adult transverse colon<br>Adult ureter<br>Adult uterus<br>Chorionic villus<br>Cord blood<br>Cord blood CD34P					
in vivo<br>Human	Kuppe *et al.*<br>(Table S9)	52 human tissue samples	Cardiomyocyte<br>Fibroblast<br>Endothelial<br>vSMCs (vascular smooth muscle cells)<br>Neuronal<br>Cycling cells<br>Lymphoid<br>Mast<br>Myeloid<br>Pericyte	41,663 cells	Raw Count	10x Genomics	GSE87038	(13)
in vitro<br>iPSC	Friedman *et al.*	Day 0, 2, 5, 15, 30	Core	42,753 cells	Raw Count	10x Genomics	E-MTAB-6268	(1)
			Proliferative					
			Early_Primed					

			Late_Primed					
			Mesendoderm					
			Definitive_Endoderm					
			Mesoderm					
			Cardiomyocyte_Precursor					
			Definitive_Endoderm					
			Cardiovascular_Progenitor					
			Mixed_Cell_Types					
			Committed_Cardiomyocyte					
			Non-Contractile					
in vitro iPSC	Shen *et al.* (Timeline)	Day 2, 3, 4, 5, 6, 7, 8, 9	Posterior Foregut	47,296 cells	Raw Count	10x Genomics	Available upon request	(14)
			Anterior Foregut					
			Cardiac Progenitors					
			Primitive Gut Endoderm					
			Paraxial and Lateral Plate Mesoderm					
			Cardiomyocytes					
			Mesendoderm					
			Definitive Endoderm					
			Endothelium					
			Axial Mesoderm					
in vitro iPSC	Shen *et al*. (Perturbation)	Day 2, 5, 9	Primitive gut endoderm	48,526 cells	Raw Count	10x Genomics	Available upon request	(14)
			Mesendoderm					
			Mesendoderm derivatives					
			Early mesendoderm					

			Anterior foregut					
			Posterior foregut					
			Cardiac lineage					
			Liver bud					
			Neural crest-like cells					
			Ciliated cells					
			Endocardial endothelium					

Table S10. Software details.

SOFTWARE	SOURCE	IDENTIFIER
R (v3.6.0 and v4.0.4)	n/a	www.r-project.org
Python (v3.8.2)	n/a	www.python.org
Seurat (v3.0)	(15)	www.satijalab.org/seurat/, https://github.com/satijalab/seurat/releases/tag/v3.0.0
scran (v1.24.1)	(16)	https://bioconductor.org/packages/release/bioc/html/scran.html
scater (v1.24.0)	(17)	https://github.com/jimhester/scater
SingleCellExperiment (v1.20.0)	(18)	https://bioconductor.org/packages/release/bioc/html/SingleCellExperiment.html
Nebulosa (v0.99.92)	(19)	https://github.com/powellgenomicslab/Nebulosa
topGO (v2.42.0)	(20)	https://bioconductor.org/packages/release/bioc/html/topGO.html
pcaExplorer (v2.9.6)	(21)	https://github.com/federicomarini/pcaExplorer
pheatmap (v1.0.12)	(22)	https://github.com/raivokolde/pheatmap
circlize (v0.4.13)	(23)	https://jokergoo.github.io/circlize_book/book/
mclust (v5.4.10)	(24)	https://mclust-org.github.io/mclust
scipy (v1.7.1)	(25)	www.scipy.org
scikit-learn (v0.22)	(26)	www.scikit-learn.org
MAGIC (v3.0.0)	(27)	https://github.com/KrishnaswamyLab/MAGIC
SC3 (v1.26.2)	(28)	https://github.com/hemberg-lab/SC3

Reference

1. Friedman, C.E., Nguyen, Q., Lukowski, S.W., Helfer, A., Chiu, H.S., Miklas, J., Levy, S., Suo, S., Han, J.-D.J., Osteil, P. *et al.* (2018) Single-Cell Transcriptomic Analysis of Cardiac Differentiation from Human PSCs Reveals HOPX-Dependent Cardiomyocyte Maturation. *Cell Stem Cell*, **23**, 586-598.e588.
2. Cao, J., Spielmann, M., Qiu, X., Huang, X., Ibrahim, D.M., Hill, A.J., Zhang, F., Mundlos, S., Christiansen, L., Steemers, F.J. *et al.* (2019) The single-cell transcriptional landscape of mammalian organogenesis. *Nature*, **566**, 496-502.
3. Lescroart, F., Wang, X., Lin, X., Swedlund, B., Gargouri, S., Sànchez-Dànes, A., Moignard, V., Dubois, C., Paulissen, C., Kinston, S. *et al.* (2018) Defining the earliest step of cardiovascular lineage segregation by single-cell RNA-seq. *Science*, **359**, 1177-1181.
4. Li, G., Xu, A., Sim, S., Priest, James R., Tian, X., Khan, T., Quertermous, T., Zhou, B., Tsao, Philip S., Quake, Stephen R. *et al.* (2016) Transcriptomic Profiling Maps Anatomically Patterned Subpopulations among Single Embryonic Cardiac Cells. *Dev Cell*, **39**, 491-507.
5. DeLaughter, D.M., Bick, A.G., Wakimoto, H., McKean, D., Gorham, J.M., Kathiriya, I.S., Hinson, J.T., Homsy, J., Gray, J., Pu, W. *et al.* (2016) Single-Cell Resolution of Temporal Gene Expression during Heart Development. *Dev Cell*, **39**, 480-490.
6. Pijuan-Sala, B., Griffiths, J.A., Guibentif, C., Hiscock, T.W., Jawaid, W., Calero-Nieto, F.J., Mulas, C., Ibarra-Soria, X., Tyser, R.C.V., Ho, D.L.L. *et al.* (2019) A single-cell molecular map of mouse gastrulation and early organogenesis. *Nature*, **566**, 490-495.
7. de Soysa, T.Y., Ranade, S.S., Okawa, S., Ravichandran, S., Huang, Y., Salunga, H.T., Schricker, A., del Sol, A., Gifford, C.A. and Srivastava, D. (2019) Single-cell analysis of cardiogenesis reveals basis for organ-level developmental defects. *Nature*, **572**, 120-124.
8. Tyser, R.C.V., Ibarra-Soria, X., McDole, K., Arcot Jayaram, S., Godwin, J., van den Brand, T.A.H., Miranda, A.M.A., Scialdone, A., Keller, P.J., Marioni, J.C. *et al.* (2021) Characterization of a common progenitor pool of the epicardium and myocardium. *Science*, **371**, eabb2986.
9. Asp, M., Giacomello, S., Larsson, L., Wu, C., Fürth, D., Qian, X., Wärdell, E., Custodio, J., Reimegård, J., Salmén, F. *et al.* (2019) A Spatiotemporal Organ-Wide Gene Expression and Cell Atlas of the Developing Human Heart. *Cell*, **179**, 1647-1660.e1619.
10. Nicin, L., Abplanalp, W.T., Schänzer, A., Sprengel, A., John, D., Mellentin, H., Tombor, L., Keuper, M., Ullrich, E., Klingel, K. *et al.* (2021) Single Nuclei Sequencing Reveals Novel Insights Into the Regulation of Cellular Signatures in Children With Dilated Cardiomyopathy. *Circulation*, **143**, 1704-1719.
11. Sim, C.B., Phipson, B., Ziemann, M., Rafehi, H., Mills, R.J., Watt, K.I., Abu-Bonsrah, K.D., Kalathur, R.K.R., Voges, H.K., Dinh, D.T. *et al.* (2021) Sex-Specific Control of Human Heart Maturation by the Progesterone Receptor. *Circulation*, **143**, 1614-1628.
12. Han, X., Zhou, Z., Fei, L., Sun, H., Wang, R., Chen, Y., Chen, H., Wang, J., Tang, H., Ge, W. *et al.* (2020) Construction of a human cell landscape at single-cell level. *Nature*, **581**, 303-309.
13. Kuppe, C., Ramirez Flores, R.O., Li, Z., Hayat, S., Levinson, R.T., Liao, X., Hannani, M.T., Tanevski, J., Wünnemann, F., Nagai, J.S. *et al.* (2022) Spatial multi-omic map of human myocardial infarction. *Nature*, **608**, 766-777.
14. Shen, S., Werner, T., Sun, Y., Shim, W.J., Lukowski, S., Andersen, S., Chiu, H.S., Xia, D., Chen, X., Pham, D. *et al.* (2022) An integrated cell barcoding and computational analysis pipeline for scalable analysis of differentiation at single-cell resolution. *bioRxiv*, 2022.2010.2012.511862.
15. Stuart, T., Butler, A., Hoffman, P., Hafemeister, C., Papalexi, E., Mauck, W.M., Hao, Y., Stoeckius, M., Smibert, P. and Satija, R. (2019) Comprehensive Integration of Single-Cell Data. *Cell*, **177**, 1888-1902.e1821.
16. Lun, A., McCarthy, D. and Marioni, J. (2016) A step-by-step workflow for low-level analysis of single-cell RNA-seq data with Bioconductor [version 2; peer review: 3 approved, 2 approved with reservations]. *F1000Research*, **5**.
17. McCarthy, D.J., Campbell, K.R., Lun, A.T.L. and Wills, Q.F. (2017) Scater: pre-processing, quality control, normalization and visualization of single-cell RNA-seq data in R. *Bioinformatics*, **33**, 1179-1186.
18. Amezquita, R.A., Lun, A.T.L., Becht, E., Carey, V.J., Carpp, L.N., Geistlinger, L., Marini, F., Rue-Albrecht, K., Risso, D., Soneson, C. *et al.* (2020) Orchestrating single-cell analysis with Bioconductor. *Nature Methods*, **17**, 137-145.
19. Alquicira-Hernandez, J. and Powell, J.E. (2021) Nebulosa recovers single-cell gene expression signals by kernel density estimation. *Bioinformatics*, **37**, 2485-2487.
20. Alexa, A. and Rahnenfuhrer, J. (2016) topGO: Enrichment analysis for Gene Ontology. R package version 2.28. 0. *Cranio*.
21. Marini, F. and Binder, H. (2019) pcaExplorer: an R/Bioconductor package for interacting with RNA-seq principal components. *BMC Bioinformatics*, **20**, 331.
22. Kolde, R. (2019).

23. Gu, Z., Gu, L., Eils, R., Schlesner, M. and Brors, B. (2014) circlize Implements and enhances circular visualization in R. *Bioinformatics*, **30**, 2811-2812.
24. Scrucca, L., Fop, M., Murphy, T.B. and Raftery, A.E. (2016) mclust 5: Clustering, Classification and Density Estimation Using Gaussian Finite Mixture Models. *R j*, **8**, 289-317.
25. Virtanen, P., Gommers, R., Oliphant, T.E., Haberland, M., Reddy, T., Cournapeau, D., Burovski, E., Peterson, P., Weckesser, W., Bright, J. *et al.* (2020) SciPy 1.0: fundamental algorithms for scientific computing in Python. *Nat Methods*, **17**, 261-272.
26. Pedregosa, F. (2011) Scikit-learn: Machine Learning in Python. *Journal of Machine Learning Research*, **12**, 2825-2830.
27. van Dijk, D., Sharma, R., Nainys, J., Yim, K., Kathail, P., Carr, A.J., Burdziak, C., Moon, K.R., Chaffer, C.L., Pattabiraman, D. *et al.* (2018) Recovering Gene Interactions from Single-Cell Data Using Data Diffusion. *Cell*, **174**, 716-729.e727.
28. Kiselev, V.Y., Kirschner, K., Schaub, M.T., Andrews, T., Yiu, A., Chandra, T., Natarajan, K.N., Reik, W., Barahona, M., Green, A.R. *et al.* (2017) SC3: consensus clustering of single-cell RNA-seq data. *Nature Methods*, **14**, 483-486.
